# Effect of low-density polyethylene microplastics on incidence of cotton Verticillium wilt and the structure and function of rhizosphere microbial community

**DOI:** 10.1186/s40793-026-00943-2

**Published:** 2026-08-01

**Authors:** Wei-song Zhao, Na-qi Cui, Xiu-yun Lu, Xiao-yun Zhang, She-zeng Li, Qing-gang Guo, Ping Ma

**Affiliations:** 1 Plant Protection Institute, Hebei Academy of Agriculture and Forestry Sciences, 437 Dongguan Street, Lianchi District, Baoding, 071000 Hebei Province China; 2 IPM Innovation Center of Hebei Province/ International Science and Technology Joint Research Center on IPM of Hebei Province, Baoding, 071000 China

**Keywords:** Low-density polyethylene, Cotton verticillium wilt, Microbial community, Metabolic, Metagenomic

## Abstract

Microplastics have become a global environmental concern, but the impact of microplastics on plant disease remains poorly understood. The primary objective of this study was to evaluate the effects of low-density polyethylene (LDPE) microplastics on cotton Verticillium wilt (CVW), as well as the structure and function of the soil microbial community. Metagenomic sequencing was performed to characterize the microbial community composition of the cotton rhizosphere. The experiment consisted of five treatments: a control group, and four LDPE groups with concentrations set at 0.1%, 0.5%, 1.0%, and 2.5%, respectively. The results demonstrated that high-dose LDPE treatments reduced the cotton emergence rate by 1.47–4.41% and plant biomass by 14.58–34.64%. These findings suggest that high-dose LDPE may exert pronounced phytotoxic effects on cotton plants. Although the populations of *Verticillium dahliae* did not differ significantly across treatments, LDPE microplastics at concentrations of 0.5–2.5% significantly exacerbated CVW, elevating the disease index by 54.29–71.67%. Statistical analysis revealed that LDPE microplastics altered the contents of soil nitrate nitrogen (NO_3_^−^-N) and available phosphorus (AP), with NO_3_^−^-N increasing by 16.86%-85.63% and AP by 51.16–69.77%. Meanwhile, LDPE microplastics triggered substantial alterations in the structure of rhizosphere microbial communities, as well as in their functional potential (assessed via KEGG pathway annotation). Microbial community structure and KEGG pathways were both significantly changed by soil nutrients (NO_3_^−^-N, NH_4_^+^-N, AP, AK). Moreover, partial least squares path modeling (PLS-PM) suggested that LDPE treatment indirectly influenced DI through two pathways: one was a positive effect mediated by alternations in fungal community structure and function, and the other was a negative effect driven by changes in soil nutrients. The exacerbation of CVW was closely linked to LDPE-induced dysbiosis in the cotton rhizosphere microenvironment.

**Graphical abstract:**

**Figure:**
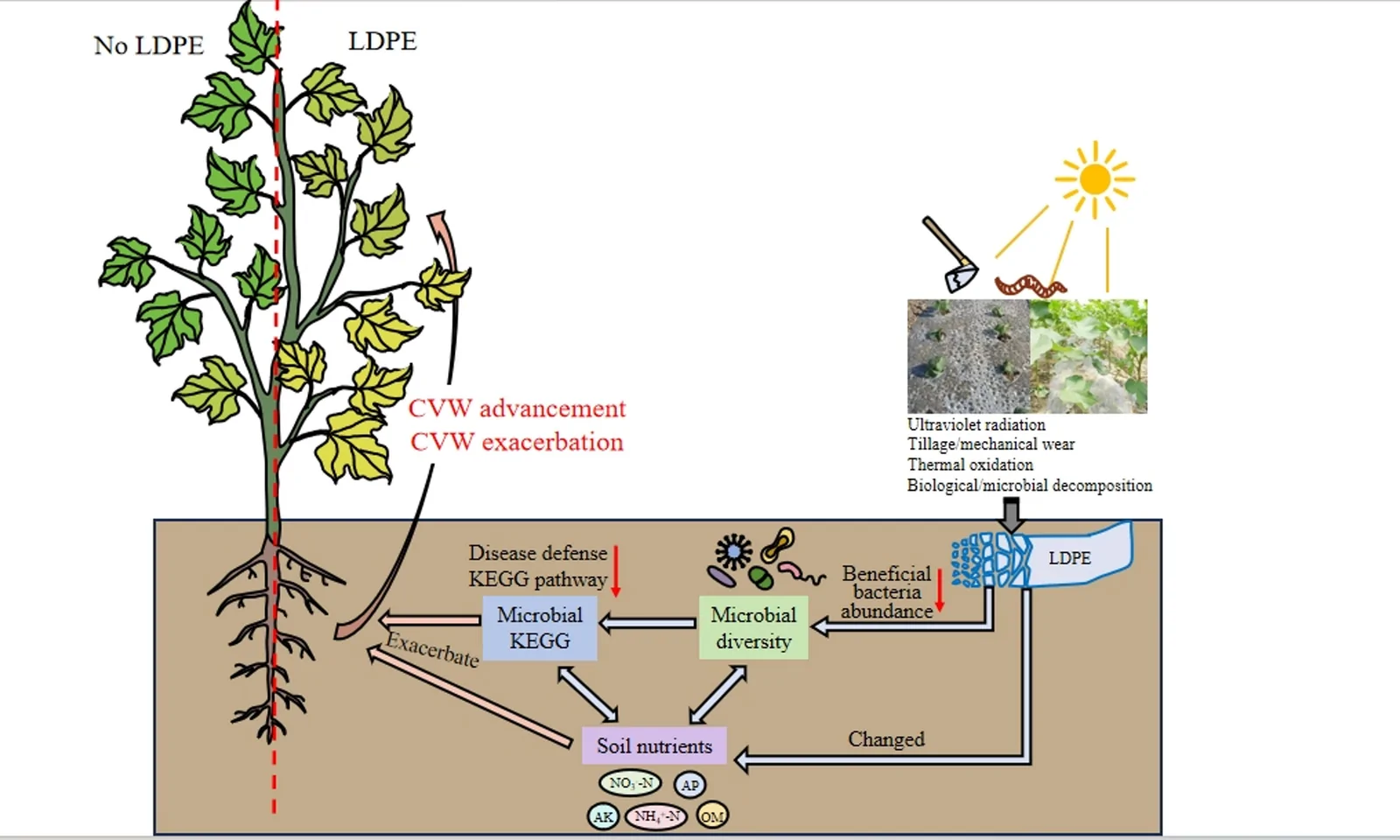

**Supplementary Information:**

The online version contains supplementary material available at https://doi.org/10.1186/s40793-026-00943-2.

## Highlights

- LDPE treatment significantly increased disease index of CVW.
- Bacterial community structure was changed in LDPE treatment.
- LDPE did not significantly change *Verticillium dahliae* populations.
- Relative abundances of major KEGG pathways of bacterial community were decreased.
- The exacerbation of CVW may be attributed to disruption of microbial community structure and function.

**Supplementary Information:**

The online version contains supplementary material available at https://doi.org/10.1186/s40793-026-00943-2.

## Introduction

Mulch is widely used in agricultural production that can improve the capacity of crop yield and has good economic benefits [1]. Due to the widespread use of plastic mulching, drip-irrigation and other agricultural activities, microplastics (MPs) have been widely detected in many agricultural soils around the world [2]. MPs characterized by their exceptionally large interfacial area, ultrafine particulate morphology, and environmental persistence, are readily assimilated or absorbed by both flora and soil-dwelling organisms, posing significant threats to agroecosystem stability and public health [3]. According to Li et al. [4] risk level of microplastics in the main agricultural areas in Xinjiang is grade III, and the abundance ranges from 288 to 1452 items/kg. Concomitantly, MPs have been detected in cotton fields in arid northwest China, with an average abundance of 28.00–1426.67 items/kg, where polyethylene (PE) agricultural films were identified as the primary source of microplastic contamination [5].

As the predominant source of natural textile fibers, cotton holds immense economic significance [6]. However, cotton production is severely impacted by Verticillium wilt, a disease that reduces crop yield and degrades fiber quality across many cotton-growing regions, with particularly severe effects in China [7]. The soil-borne pathogen *Verticillium dahliae*, whose resilient microsclerotia maintain viability for over a decade without host plants or during environmental stresses, represents the primary etiological agent of cotton Verticillium wilt (CVW) [8].

Numerous studies have investigated the impacts of MPs on soil ecosystem, including soil physicochemical properties, enzyme activity, the composition and metabolic functions of soil microbial communities, and plant growth [9–13]. For instance, Polyvinyl chloride (PVC) residues have been shown to significantly inhibited soil enzyme activities clearly [14]. Exposure to low-density polyethylene (LDPE) decreased the abundance of 14 bacterial genera, including *Sphingomonas*, disrupted bacterial co-occurrence networks, and altered the spectrum of soil metabolites [15]. PE has also been reported to influence microbial diversity and function profiles, potentially enriching pathogenic microorganisms [16]. Furthermore, PE was found to substantially restrict root growth and significantly reduced seed cotton yield [5]. Exposure to PVC resulted in significant reductions in foliar surface area, impaired photosynthetic efficiency parameters, and diminished iron accumulation in plant tissues [17]. In contrast, Lozano et al. [18] observed that PE, Polyethylene terephthalate (PET) and Polypropylene (PP) all increased shoot and root mass of carrot. Both Polystyrene (PS) microplastics and nanoplastics disturb earthworm metabolism, with PS nanoplastics reducing the diversity and abundance of gut microbiota [19, 20].

Despite these findings, there is limited understanding of how LDPE affect soil-borne disease. Thus, it is imperative to investigate the ecological mechanisms through which LDPE application affects CVW, soil nutrients and microbial community within plant–microbe-soil systems. The objectives of this study were to (1) explore the impact of LDPE on CVW and cotton plant growth; (2) reveal effects of LDPE on soil nutrients, structure and function of microbial communities; (3) identify potential pathways through which LDPE influences CVW development.

## Materials and methods

### Materials, pathogen and plant variety

Low-density polyethylene (LDPE) was purchased from Dongguan Shengbang Polymer Co., Ltd (Guangdong, China). The particles had a mean diameter is 150 µm, a density is 0.913 g/cm^3^, and a purity was 98%. A susceptible cotton variety, Ejing No. 1 (EJ1), and the soil-borne pathogen *V. dahliae* Vd991, the causal agent of cotton Verticillium wilt (CVW), were used in this study.

### Experimental design

Vd991 was first reactivated on potato dextrose agar plates at 25 °C for 7 d. Subsequently, five mycelial plugs (5 mm diameter) were inoculated into a 300 mL Erlenmeyer flask containing 200 mL of CM (Complete Medium) liquid medium and incubated at 25 °C with shaking at 180 rpm for 4 d. The concentration of the resulting conidial suspension was determined using a hemocytometer and adjusted to 5.0 × 10^7^ spores/mL. This suspension was then thoroughly mixed into the soil to achieve a final concentration of 10^6^ spores per gram of dry soil. After sterilization in 3% NaClO (1 min) and rinsing, EJ1 seeds were germinated on moist gauze, then transferred to pots (12 cm × 15 cm × 14 cm) containing various LDPE treatments. The LDPE concentrations were 0.1%, 0.5%, 1.0% and 2.5% (w/w), the treatment without LDPE was the control (CK). All treatments except CK were inoculated with *V. dahliae* suspension, while CK received the same volume of sterile medium. Ten seeds were contained in per pot. All treatments were cultured in a greenhouse at 20–25 ℃, with a 14 h light and 10 h dark photoperiod and relative humidity of 55–65%.

### Soil sample collection and assessment of plant health among different LDPE treatments

CVW was investigated at 30 d and 60 d, respectively. Plant height, ground diameter, fresh weight and dry weight were investigated at 60 d. The rhizosphere soil was collected at 30 d and 60 d after planting. Briefly, for each treatment, five pots were randomly selected, and the root systems of all plants (50 plants total) were excavated. After gently shaking to remove loosely adhered soil, the rhizosphere soil attached to the root was collected by brushing. From the 50 plants, three independent composite replicates were prepared, each consisting of rhizosphere soil pooled from 15 randomly selected plants (45 plants in total for the three replicates). The composite samples were sieved (40-mesh, 0.425 mm) to remove fine roots, then subdivided: − 80 °C for metagenomics (n = 3) and − 20 °C for soil nutrients/*V. dahliae* ITS gene copy numbers (n = 3).

The severity of CVW was assessed at 30 and 60 d using a disease index (DI) on a 0–4 scale [21]: 0 = healthy plant, no diseased leaves; 1 = plant with 25% leaves showing infection symptoms; 2 = plant with 25%-50% leaves showing symptoms); 3 = plants exhibiting symptoms on 50% to 75% of their leaves; and 4 = severe infection (> 75% leaf coverage) or complete plant death

$$ \begin{aligned} & DI \\ & = \sum {\frac{{{\mathrm{No}}.\,{\mathrm{of}}\,{\mathrm{diseased}}\,{\mathrm{plants}} \times {\mathrm{corresponding}}\,{\mathrm{disease}}\,{\mathrm{rating}}}}{{4 \times {\mathrm{total}}\,{\mathrm{plant}}\,{\mathrm{numbers}}}}} \times 100 \\ \end{aligned} $$

The plant was dug up and the root was cleaned. Plant height, ground diameter, fresh weight and dry weight were compared in different LDPE treatments at 60 d.

### Soil DNA extraction and populations of *V. dahliae* in soil by qPCR

Total genomic DNA was extracted from soil samples using the FastDNA™ SPIN Kit (MP Biomedicals, Solon, OH, USA) according to the manufacturer's instructions. DNA quantification and quality control were performed on a NanoDrop 2000 instrument (Thermo Fisher Scientific, MA). qPCR was employed to measure *V. dahliae* DNA copy numbers [22], as detailed below.

The ITS region of *V. dahliae* was amplified with primers DB19 (5′-CGGTGACATAATACTGAGAG-3′) and DB20 (5′-GACGATGCGGGATTGAACGAA-3′), cloned into pMD19-T, and sequenced, confirming 100% homology to the ITS gene. The recombinant plasmid was used to generate a standard curve. qPCR was performed with primers F (5′-CCCGCCGGTCCATCAGTCTCTCTCTG-3′) and R (5′-CGGGACTCCGATGCGAGCTGTAAC-3′), and Carboxy‑X‑rhodamine as reference dye. The 20‑μL reaction contained 10 μL 2 × Master Mix, 1 μL each primer, 0.4 μL Carboxy‑X‑rhodamine, and 1 μL template. A standard curve was generated using tenfold serial dilutions of the plasmid, plotting Ct values against log copy numbers. *V. dahliae* copy numbers in samples were calculated from the standard curve based on average Ct of triplicates.

### Metagenomic sequencing and bioinformatic analysis

Metagenomic sequencing was conducted on an Illumina HiSeq platform (PE 300) by Majorbio Biopharm Technology Co., Ltd (Shanghai, China). Fastp v0.20.0 was used for quality control of raw data. Removing low-quality and N-containing reads to obtain high-quality sequences. Assembled contigs were binned by MEGAHIT v1.1.2, and select 300 bp continuous sequences for further gene prediction and annotation. Sequencing data was clustered by CD-HIT and a non-redundant gene set is constructed. Annotate the species by DIAMOND to obtain the abundance of each species at each taxonomy level in different treatments. Functional annotation was performed against the KEGG databases using DIAMOND.

### Assessment of soil nutrients in different LDPE treatments

Air-dried soil served for determining pH, organic matter (OM), available phosphorus (AP), and available potassium (AK), with fresh soil reserved for ammonium nitrogen (NH_4_^+^-N) and nitrate nitrogen (NO_3_^−^-N) contents based on previous methodologies [23, 24].

### Statistical analysis

Significant differences among treatments (*P* < 0.05) were identified using Duncan's multiple range test in SPSS 17.0. Principal coordinates analysis (PCoA) based on the Bray–Curtis distance was performed using the vegan package in R. Bubble plots illustrating different KEGG pathways were generated using the ggplot2 package in R Studio. Redundancy analysis (RDA) was conducted using CANOCO 5.0 to explore the relationships between soil nutrients and microbial community structure. Mantel test and Partial least squares path modeling (PLS-PM) were used to determine the relationship between microbial community, soil nutrients and DI by package of linkET and plspm respectively in R Studio.

## Results

### Effects of different LDPE treatments on CVW, plant growth and *V. dahliae* in soil

**Fig. 1:**
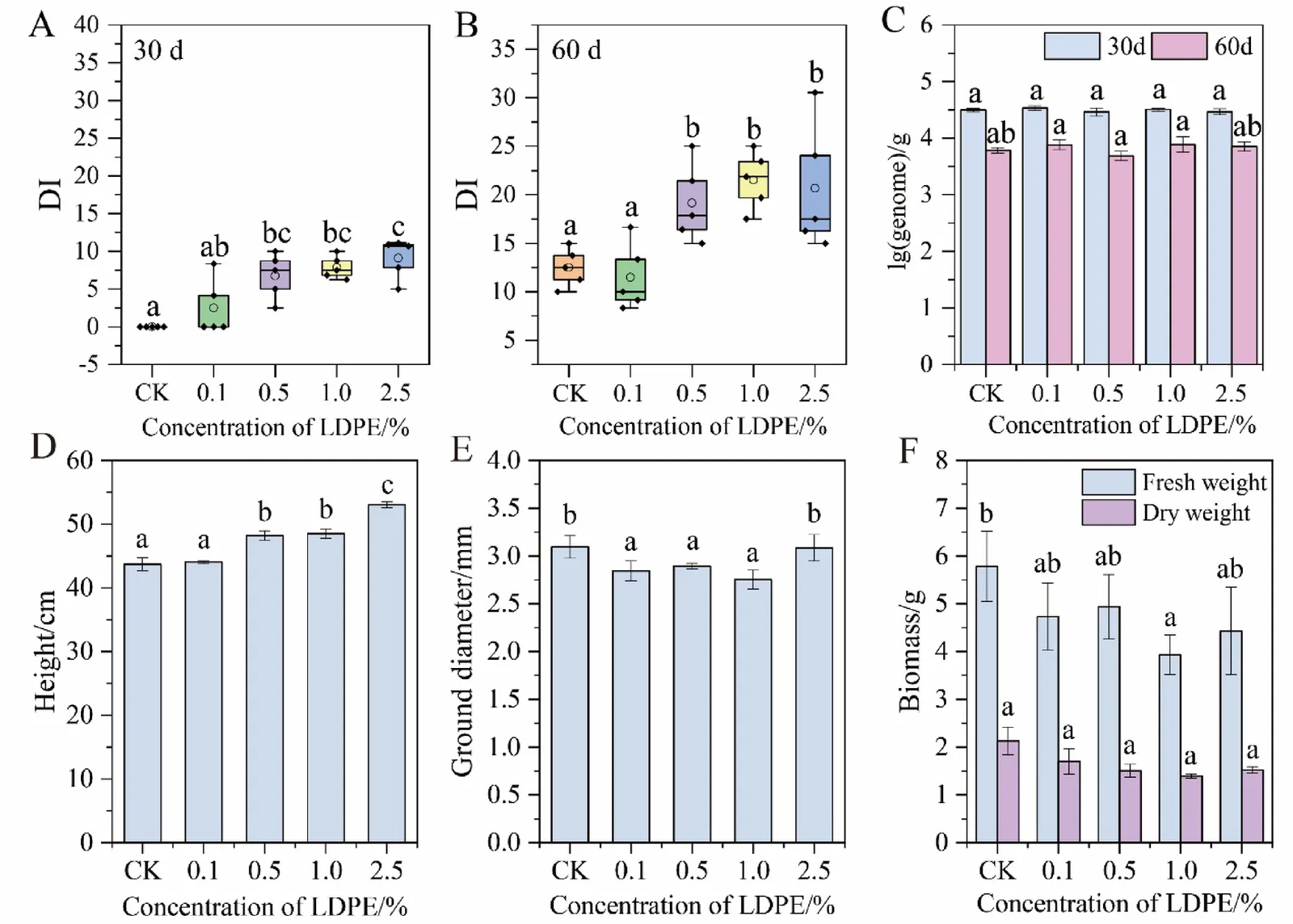
Effect of different LDPE treatments on **A** DI of CVW at 30d, **B** DI at 60d, **C** *V. dahliae* DNA copy numbers in soil, **D** Height, **E** Ground diameter and **F** Biomass. Different letters indicate significant difference among treatments by Duncan’s multiple range test at *P* < 0.05. *Note*: LDPE, low-density polyethylene; DI, disease index; CVW, cotton Verticillium wilt; CK, control

At 30 d, the DI reached 2.78, 6.67, 7.92 and 8.94 under LDPE treatments with 0.1%, 0.5%, 1.0% and 2.5%, respectively, whereas no disease was observed in CK treatment (Fig. 1A). Moreover, at 60 d, the DI increased to 11.50, 19.28, 21.46 and 21.02 under LDPE treatment with 0.1%, 0.5%, 1.0% and 2.5%, respectively (Fig. 1B). Compared with CK (DI = 12.50), the DI at 60 d under 0.5%, 1.0% and 2.5% LDPE treatments significantly increased by 54.29%, 71.67% and 68.15%, respectively. However, no significant treatment effects on *V. dahliae* populations were observed (Fig. 1C).

Cotton emergence rates on 6–8 d were reduced in all LDPE treatments, though differences among concentrations were not statistically significant (Table S1). Plant height significantly increased by 10.31%, 11.01% and 21.37% respectively, when LDPE concentrations were 0.5%, 1% and 2.5% (Fig. 1D). In contrast, ground diameter significantly decreased by 8.17%, 6.48% and 11.05% with the LDPE concentrations at 0.1%, 0.5% and 1% respectively (Fig. 1E). Fresh weight was significantly decreased by 31.98% in 1.0% LDPE treatment, while dry weight remained unaffected across all treatments (Fig. 1F).

### Effects of different LDPE treatments on soil nutrients

At 30 d, compared with CK, OM contents were significantly increased by 50.67% and 85.33% in 1.0% and 2.5% LDPE treatments, respectively. NO_3_^−^-N contents were significantly decreased by 48.65% and 21.62% in 0.1% and 1.0% LDPE, respectively, but increased by 22.52% in 0.5% LDPE treatments, respectively. AK contents were significantly decreased by 69.14% and 84.74% in 0.5% and 1.0% LDPE treatment, respectively. pH in 0.5% LDPE treatment was significantly higher than CK, while no significant differences were observed in NH_4_^+^-N or AP contents among treatments.

By 60 d, compared with CK, OM contents remained significantly increased in 1.0% and 2.5% LDPE treatments, with increases of 16.70% and 51.67%, respectively. AP contents were significantly increased by 69.77%, 60.47% and 51.16% in 0.1%, 0.5% and 1.0% LDPE treatments, respectively. LDPE treatments did not significantly affect NH_4_^+^-N and AK contents (Table 1).

**Table 1:** LDPE effects on soil nutrients

Days	Index	Soil nutrients under different concentrations of LDPE (mean ± SD)				
		CK	0.1%	0.5%	1.0%	2.5%
30 d	pH	7.55 ± 0.08 a	7.64 ± 0.02 ab	7.70 ± 0.02 b	7.67 ± 0.04 ab	7.69 ± 0.05 ab
	NO_3_^−^-N(μg/g)	6.65 ± 0.25 c	3.42 ± 0.18 a	8.15 ± 0.37 d	5.22 ± 0.54 b	7.19 ± 0.25 c
	NH_4_^+^-N(μg/g)	10.23 ± 1.50 a	12.03 ± 1.97 a	12.94 ± 1.48 a	11.40 ± 0.79 a	11.10 ± 1.05 a
	AP(μmol/g)	1.21 ± 0.21 a	1.15 ± 0.05 a	1.07 ± 0.10 a	1.00 ± 0.22 a	1.08 ± 0.02 a
	AK(mg/kg)	78.11 ± 9.26c	72.87 ± 9.79 bc	62.15 ± 7.12 ab	54.00 ± 3.73 a	66.18 ± 2.01 abc
	OM(%)	1.74 ± 0.03 a	1.82 ± 0.04 a	1.80 ± 0.03 a	2.63 ± 0.02 b	3.23 ± 0.01 c
60 d	pH	7.65 ± 0.03 ab	7.60 ± 0.01 a	7.64 ± 0.01 ab	7.66 ± 0.02 ab	7.69 ± 0.01 b
	NO_3_^−^-N(μg/g)	1.98 ± 0.15 a	4.05 ± 0.67 a	1.25 ± 0.30 a	1.62 ± 0.32 a	1.52 ± 0.32 a
	NH_4_^+^-N(μg/g)	4.38 ± 0.36 ab	4.47 ± 0.64 ab	3.07 ± 0.73 a	5.31 ± 0.90 b	4.69 ± 0.84 b
	AP(μmol/g)	0.43 ± 0.09 a	0.73 ± 0.06 b	0.69 ± 0.06 b	0.65 ± 0.57 b	0.42 ± 0.09 a
	AK(mg/kg)	97.73 ± 2.07 a	111.06 ± 3.28 a	108.68 ± 6.61 a	111.65 ± 5.04 a	100.10 ± 3.71 a
	OM(%)	2.61 ± 0.01 a	2.60 ± 0.06 a	2.80 ± 0.02 a	3.05 ± 0.02 b	3.96 ± 0.02 c

Different letters mean significant difference among all the treatments by Duncan’s multiple range test at *P* < 0.05

### Effects of different LDPE treatments on bacterial and fungal communities diversity

The Illumina sequencing generated 1,745,124,250 raw reads, with 1,712,928,332 clean reads retained after quality filtering, representing a > 97.60% clean reads across all libraries. After assembly, 5,861,481,867 bases were found in 11,777,240 contigs. Contig length distribution analysis demonstrated that ≥ 50% of sequences were longer than 495 bp (N50). The complete genes had a maximum contig length of 544,290 bp and a minimum length of 292,158 bp (Table S2).

At 30 d, no significant differences in Shannon and Chao l index were observed for either bacterial and fungal communities between the LDPE treatments and the CK. By 60 d, however, Shannon index of both bacterial and fungal community was significantly increased in LDPE treatments compared with CK. The Chao 1 index also showed an increasing trend in LDPE treatments, though the differences were not statistically significant. For fungal community, the Shannon index was higher in LDPE-treated soils, whereas the Chao1 index remained unaffected (Table 2).

**Table 2:** LDPE effects on alpha diversity of bacterial and fungal communities

Microorganism	Treatment	30d		60d	
		Shannon index	Chao 1 index	Shannon index	Chao 1 index
bacteria	CK	4.72 ± 0.01 ab	3027 ± 37.03 a	4.62 ± 0.01 a	3019.33 ± 7.57 a
	0.1%	4.70 ± 0.01 a	3030.00 ± 22.72 a	4.64 ± 0.01 b	3039.67 ± 8.50 a
	0.5%	4.71 ± 0.01 ab	3025.67 ± 15.95 a	4.66 ± 0.01 c	3026.33 ± 6.51 a
	1.0%	4.72 ± 0.01 ab	3016.33 ± 13.05 a	4.66 ± 0.01 c	3034.00 ± 10.15 a
	2.5%	4.72 ± 0.01 b	3001.00 ± 33.06 a	4.66 ± 0.01 c	3031.67 ± 17.21 a
fungi	CK	3.51 ± 0.49 a	522.67 ± 20.03 a	3.82 ± 1.41 a	498.33 ± 26.65 ab
	0.1%	3.90 ± 0.45 a	530.67 ± 8.50 a	4.58 ± 0.04 a	523.00 ± 33.51 b
	0.5%	3.95 ± 0.49 a	531.67 ± 2.08 a	4.38 ± 0.09 a	509.33 ± 12.06 ab
	1.0%	4.08 ± 0.22 a	528.33 ± 2.08 a	4.38 ± 0.03 a	472.67 ± 9.02 a
	2.5%	4.07 ± 0.21 a	518.67 ± 12.34 a	4.45 ± 0.01 a	489 ± 19.97 ab

Different letters mean significant difference among all the treatments by Duncan’s multiple range test at *P* < 0.05

**Fig. 2:**
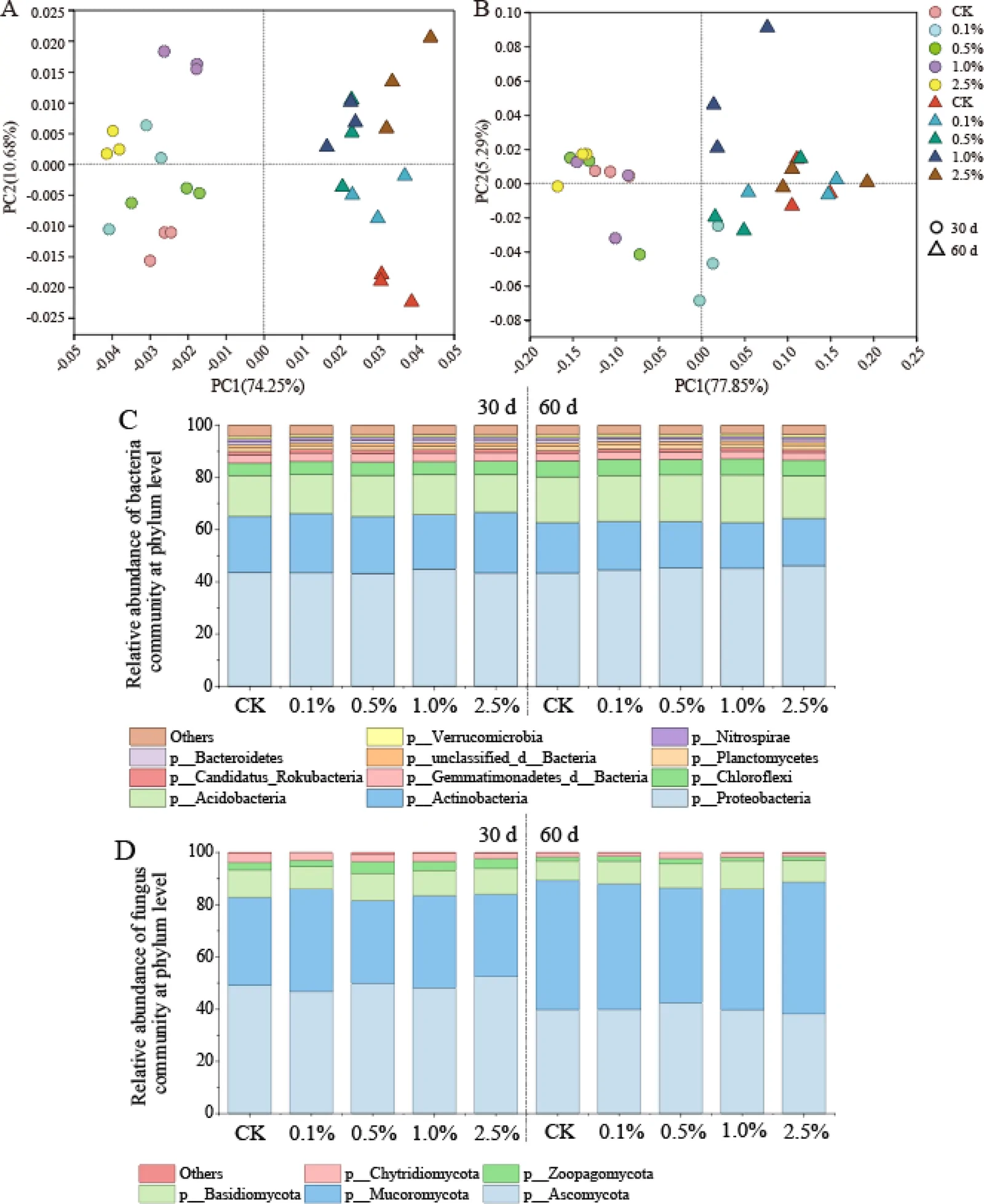
Principal coordinates analysis (PCoA) of **A** bacterial community and **B** fungal community in different LDPE treatments. LDPE effects on (**C**) bacterial community and **D** fungal community composition at the phylum level. *Note*: In (A) and (B), circles and triangles represent samples at 30 and 60 d, respectively. Colors indicate different LDPE treatments (CK, 0.1%, 0.5%, 1.0% and 2.5%). In **C** and **D**, the left and right groups of five bars correspond to 30 and 60 d, separated by a dashed line

Principal coordinate analysis (PCoA) performed at the phylum level further revealed structural shifts in soil bacterial and fungal communities under LDPE treatments (Fig. 2). For bacteria, the top two coordinates explained 84.93% of the total variation, with PC1 and PC2 contributing 74.25% and 10.68%, respectively (Fig. 2A). Clear separation along PC1 was observed between bacterial communities at 30 and 60 d. Moreover, the 0.5%, 1.0%, and 2.5% LDPE treatments were distinctly separated from the CK at both 30 and 60 d, indicating that 0.5%, 1.0%, and 2.5% LDPE treatments significantly altered bacterial community structure.

Fungal community structure was also influenced by LDPE treatments (Fig. 2B). The two primary coordinates accounted for 83.14% of the total variation, with PC1 accounting for 77.85% and PC2 accounting for 5.29%. PCoA showed an obvious separation between CK and 0.1% LDPE treatment in the fungal community structure at 30 d, and between CK and 1.0% LDPE treatment at 60 d.

### Effects of different LDPE treatments on bacterial and fungal communities composition

For the bacterial community, the dominant bacterial phyla were Proteobacteria, Actinobacteria, Acidobacteria, Chloroflexi, Gemmatimonadetes, Candidatus_Rokubacteria, Planctomycetes, Bacteroidetes, Nitrospirae and Verrucomicrobia (relative abundance (RA) in all treatments was more than 1%). Collectively, these phyla accounted for over 97% of the total bacterial sequences, with *Proteobacteria* being the most abundant (Fig. 2C). The relative abundance profiles of bacterial phyla under different LDPE treatments are presented in Table S3 and S4.

For the fungal community, the dominant fungal phyla in the cotton rhizosphere soil in different groups were *Ascomycota*, *Mucoromycota*, *Basidiomycota*, *Zoopagomycota* and *Chytridiomycota* (RA > 1%). These phyla collectively represented > 99% of the total fungal community, with *Ascomycota* being the most abundant (Fig. 2D). The relative abundance profiles of bacterial phyla under different LDPE treatments are presented in Table S5 and S6.

**Fig. 3:**
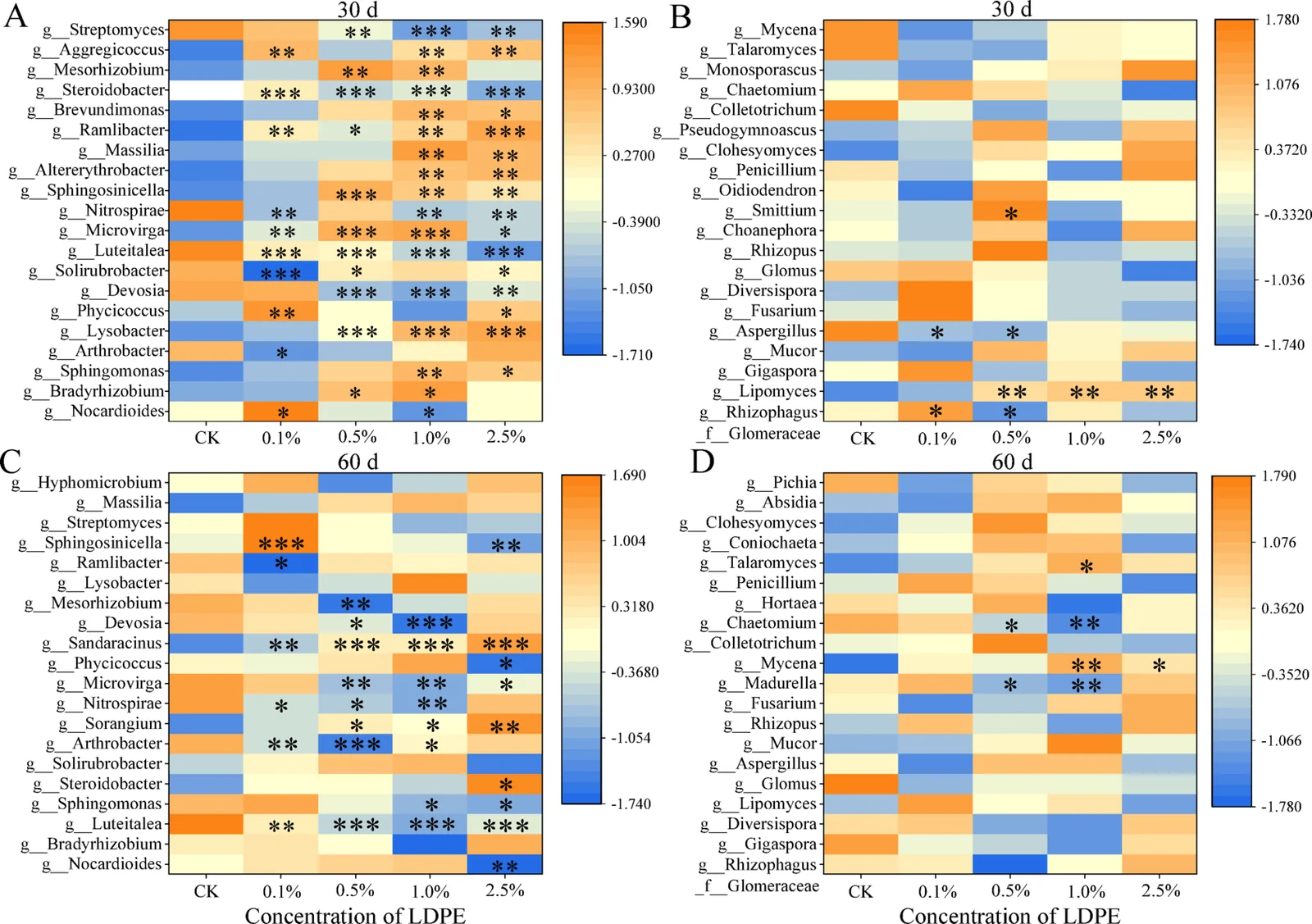
Significant analysis of microbial community abundance at the genus level. **A** Bacterial community and **B** fungal community at 30 d, **C** bacterial community and **D** fungal community at 60 d. The significant correlations with CK are presented as asterisks (*P < 0.05; **P < 0.01; ***P < 0.001)

To better elucidate bacterial population dynamics under LDPE treatments, genus-level profiling was conducted (unidentified genera of the microbial community were removed, RA > 1%). Statistical variance analysis revealed response bacterial genera showing significant proliferation or reduction in LDPE treatments at 30 d (Fig. 3A). The top 3 bacterial communities in all treatments were *Nocardiodies*, *Bradyrhizobium* and *Sphingomonas.* Compared with CK, the relative abundances *of Devosia, Luteitalea, Steroidobacter and Streptomyces* significantly decreased by 6.67%, 10.01%, 31.50% and 7.78% in 0.5% LDPE treatment, 4.51%, 15.37%, 27.97% and 12.46% in 1.0% LDPE treatment, 7.64%, 20.89%, 37.50% and 10.65% in 2.5% LDPE treatment. However, the relative abundances of *Sphingomonas, Lysobacter, Sphingosinicella, Altererythrobacter*, *Massilia*, *Ramlibacter Brevundimonas* and *Aggregicoccus* significantly increased by 117.07%, 58.00%, 26.24%, 30.53%, 64.32%, 16.91%, 125.33% and 22.06% in 1.0% LDPE treatment, 103.66%, 65.15%, 22.04%, 31.64%, 56.40%, 21.07%, 114.39% and 26.96% in 2.5% LDPE treatment. As for fungal community, compared with CK, the relative abundances of *Lipomyces* significantly increased by 61.17%, 72.56% and 71.89% in 0.5%, 1.0% and 2.5% LDPE treatment, respectively (Fig. 3B). Similarly, *Smittium* in 0.5% LDPE treatment and *Rhizophagus* in 0.1% LDPE treatment significantly increased by 67.30% and 46.56%, respectively. In contrast, the relative abundances of *Aspergillus* decreased by 30.48% and 32.47% in 0.1% and 0.5% LDPE treatments. *Rhizophagus_f__Glomeraceae* significantly decreased by 24.07% in 0.5% LDPE treatment.

At 60 d, the top 3 bacterial communities in all treatments were *Nocardiodies*, *Bradyrhizobium* and *Luteitalea* (Fig. 3C). Compared with CK, the relative abundances of *Sorangium* significantly increased by 6.43%, 5.55%, 11.34% in 0.5%, 1.0% and 2.5% LDPE treatments, while *Sandaracinus* increased by 27.41%, 27.41%, 44.96%. In contrast, *Luteitalea* significantly decreased by 6.09%, 7.33% and 5.33% under the same treatments. The relative abundances of *Arthrobacter*, *Nitrospira, Microvirga* and *Devosia* significantly decreased by 18.81%, 17.32%, 7.47% and 6.64% in 0.5% LDPE treatment, 6.73%, 21.13%, 8.39% and 14.06% in 1.0% LDPE treatment. Certain genera decreased significantly under specific LDPE treatments: the relative abundances of *Nocardioides, Sphingomonas, Microvirga, Phycicoccus* and *Sphingosinicella* significantly decreased by 12.85%, 9.86%, 5.57%, 11.05% and 5.20% in 2.5% LDPE treatment. As for fungal community, the top 3 genus in 0.1%, 0.5% and 1.0% LDPE treatments were *Rhizophagus_f__Glomeraceae*, *Gigaspora* and *Lipomyces*, while for CK and 2.5% LDPE treatments were *Rhizophagus_f__Glomeraceae*, *Gigaspora* and *Diversispora* (Fig. 3D). Significant changes in the relative abundance of other fungal genera were also observed: *Madurella* and *Chaetomium* significantly decreased by 46.48% and 42.17% in 0.5% and 1.0% LDPE treatments, while *Mycena* significantly increased by 151.28% and 114.00% in 1.0% and 2.5% LDPE treatments. In addition, the relative abundance of *Talaromyces* significantly increased by 104.57% in 1.0% LDPE treatment.

### Effects of different LDPE treatments on KEGG pathways of bacterial and fungal communities

**Fig. 4:**
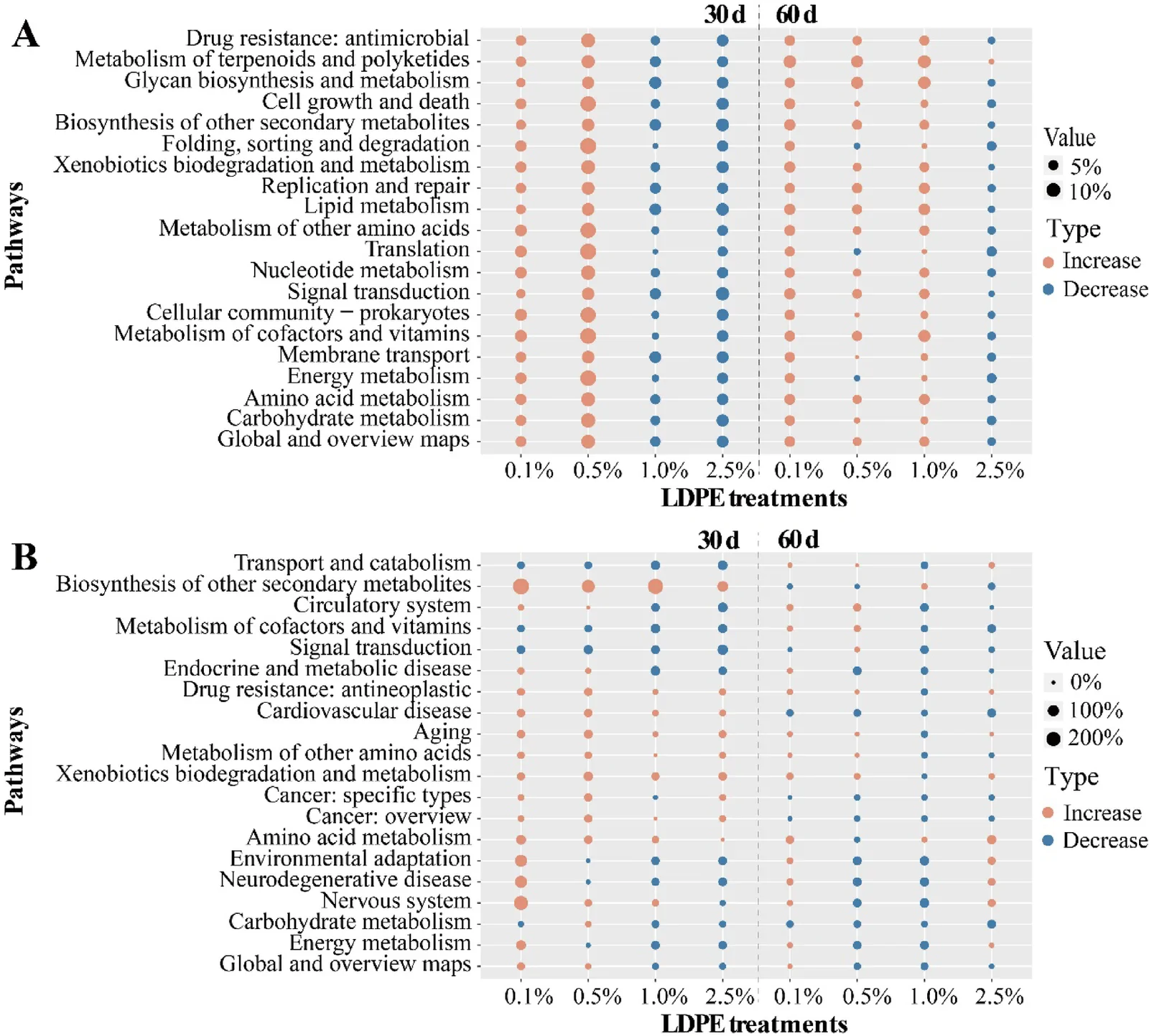
Effect of different LDPE treatments on KEGG level 2 pathways in **A** bacterial community and **B** fungal community. Red and blue bubbles indicated the relative increase or decrease in LDPE treatment compared to CK, respectively. The size of the bubble indicates the percentage of the increase or decrease

The top 20 KEGG level 2 pathways were analyzed to evaluate functional changes in bacterial and fungal communities under LDPE treatments (Fig. 4). In the bacterial community, the dominant functional categories at both 30 and 60 d included global and overview maps (27.33% and 27.19%), carbohydrate metabolism (9.45% and 9.50%), amino acid metabolism (8.77% and 8.71%), energy metabolism (6.48% and 6.46%), metabolism of cofactors and vitamin (4.52% and 4.44%), membrane transport (4.47% and 4.47%), cellular community-prokaryotes (3.79% and 3.81%) and signal transduction (3.42% and 3.51%) (Table S7). At 30 d, compared with CK, the abundances of most top 20 KEGG pathways were increased in 0.1% LDPE and 0.5% LDPE treatments but decreased in 1.0% LDPE and 2.5% LDPE treatments, such as carbohydrate metabolism (decreased by 3.73% and 7.11%), amino acid metabolism (decreased by 4.05% and 5.93%), energy metabolismand (decreased by 1.38% and 5.43%) and membrane transport (decreased by 6.38% and 6.68%) (Fig. 4A). At 60 d, the abundances of most pathways (except for energy metabolism, translation, folding, sorting and degradation) increased in 0.1%, 0.5% and 1.0% LDPE treatments relative to CK. In contrast, 2.5% LDPE treatment led to widespread decreases, including carbohydrate metabolism (decreased by 3.32%), amino acid metabolism (decreased by 2.07%), energy metabolismand (decreased by 3.62%) and membrane transport (decreased by 2.69%).

For fungal community, the dominant KEGG level 2 functional categories at 30 d and 60 d included global and overview maps (21.92% and 20.12%), energy metabolism (8.17% and 9.53%), carbohydrate metabolism (6.86% and 5.84%), nervous system (5.72% and 6.51%), neurodegenerative disease (5.58% and 7.24%), environmental adaptation (5.54% and 7.51%) (Table S7). At 30 d, most of top 20 KEGG pathways showed increased gene abundances in 0.1% and 0.5% LDPE treatments compared with CK. In contrast, the gene abundances related to energy metabolism (decreased by 47.01% and 41.03%), carbohydrate metabolism (decreased by 32.69% and 20.19%), neurodegenerative disease (decreased by 35.71% and 45.71%) and environmental adaptation (decreased by 41.73% and 45.71%) in 1.0% and 2.5% LDPE treatments were decreased. Pathways such as amino acid metabolism (increased by 26.09% in 1.0% treatment), xenobiotics biodegradation and metabolism (increased by 60.00% and 37.14%), metabolism of other amino acids (increased by 18.42% in 2.5% treatment) and aging (increased by 8.82% and 29.41%) were increased in these higher LDPE treatments. By 60 d, 0.1% LDPE treatment still increased the abundance of most gene abundances compared with CK, whereas 0.5% and 1.0% LDPE treatments generally decreased them, such as energy metabolism (decreased by 40.84% and 48.70%), carbohydrate metabolism (decreased by 28.03% and 17.20%), nervous system (decreased by 43.42% and 61.18%), environmental adaptation (decreased by 47.40% and 50.38%).

### Relationships between soil nutrients and microbial community composition and functional pathways

**Fig. 5:**
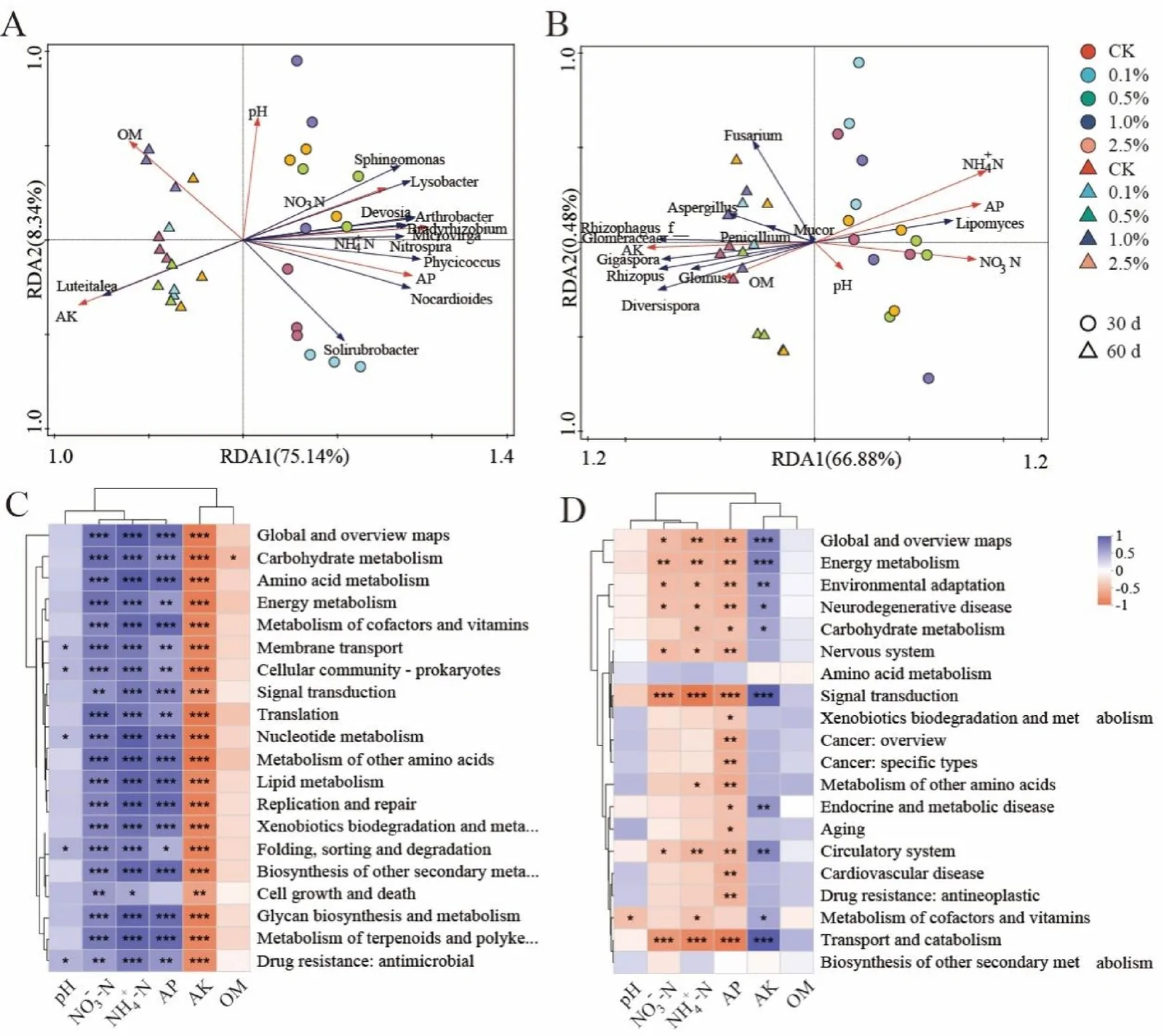
RDA of correlations between microbial community and soil nutrients in different LDPE treatments: **A** bacterial community and **B** fungal community. Heatmap of relationships between KEGG level 2 pathways and soil nutrients according to the result of Spearman’s correlation: **C** bacterial community and **D** fungal community. Positive relationships are represented in blue, while negative relationships are represented in red. The significant correlations are presented as asterisks (*P < 0.05; **P < 0.01; ***P < 0.001). *Note*: In **A** and **B**, circles and triangles represent samples at 30 and 60 d, respectively; colors indicate different LDPE treatments. In **C** and **D**, AP, available potassium; AK, available phosphorus; OM, organic matter

The RDA revealed distinct relationships between soil nutrients and microbial communities under LDPE treatments (Fig. 5). For bacterial community, RDA1 and RDA2 together explained 83.48% of the total variation (75.14% and 8.34% respectively) (Fig. 5A), while for fungal community, the cumulative explanation was 67.36% (66.88% and 0.48%) (Fig. 5B). Furthermore, significant correlations between soil nutrients and microbial community composition were clarified by determining the r^2^ and *P* values (Table S8). In bacterial community, pH (r^2^ = 0.2835, *P* = 0.018), NO_3_^−^-N (r^2^ = 0.5763, *P* = 0.001), NH_4_^+^-N (r^2^ = 0.8711, *P* = 0.001), AP (r^2^ = 0.7763, *P* = 0.001), AK (r^2^ = 0.7306, *P* = 0.001) and OM (r^2^ = 0.4831, *P* = 0.035) all exhibited significant correlations with community. Similarly, in fungal community, NO_3_^−^-N (r^2^ = 0.5225, *P* = 0.001), NH_4_^+^-N (r^2^ = 0.7861, *P* = 0.001), AP (r^2^ = 0.5904, *P* = 0.001), AK (r^2^ = 0.6456, *P* = 0.001) and OM (r^2^ = 0.2184, *P* = 0.031) were significantly associated with structure. These results suggested that NO_3_^−^-N, NH_4_^+^-N, AP, AK and OM were key factors significant shaping both bacterial and fungal community in LDPE treatments.

Spearman correlation analysis was performed to assess the relationships between the top 20 abundant KEGG level 2 pathways and soil nutrients (Fig. 5C and D). In bacterial community, most pathways, including carbohydrate metabolism, amino acid metabolism, energy metabolism and signal transduction, exhibited significant positive correlations with NO_3_^−^-N, NH_4_^+^-N and AP, but were negatively correlated with AK (Fig. 5C). In contrast, for fungal community, the majority of pathways showed significant negative correlations with AP. Furthermore, energy metabolism, environmental adaptation, neurodegenerative disease, signal transduction, circulatory system, transport and catabolism showed significant negative correlations with NO_3_^−^-N and NH_4_^+^-N, while positive correlations with AK (Fig. 5D).

### Effects of different LDPE treatments on nitrogen and sulfur cycling of cotton rhizosphere soil

Based on KEGG annotation results, functional genes and enzymes associated with nitrogen and sulfur cycle were analyzed. The analysis revealed that multiple functional genes encoding distinct enzymes mediated nitrogen and sulfur transformations in cotton rhizosphere soils under different LDPE concentrations. In the nitrogen cycle, at 30 d, the abundances of nitrification-related genes including *Hao* and *AmoC,* decreased in LDPE treatments compared with CK, while *AmoB* abundance increased (Fig. S1A). For dissimilatory nitrate reduction, the abundances of *NapAB*, *NirB* and *NrfAH* decreased in 1.0% and 2.5% LDPE treatments, respectively. At 60 d, the abundances of nitrification-related genes, such as *NarB*, *Hao* and *AmoABC,* decreased in 0.5%, 1.0% and 2.5% LDPE treatments, respectively (Fig. S1B). Denitrification genes including *NapB*, *NisS* and *NorC* abundances also decreased under these treatments. In contrast, other genes involved in the denitrification pathway, including *NapA*, *NorB* and *NosZ,* showed increased abundances across the corresponding LDPE treatments. Similarly, *NapA*, *NirBD* and *NrfAH* of dissimilatory nitrate reduction increased in different LDPE treatments. Collectively, most genes associated with nitrogen cycling exhibited more significant changes in the 0.5%, 1.0% and 2.5% LDPE treatments compared to 0.1% LDPE treatment.

In the sulfur cycle, at 30 d, the abundances of *CysCDHJN* and *Sir* involved in assimilatory sulfate reduction decreased in 1.0% and 2.5% LDPE treatments, respectively, compared with the CK (Fig. S1C). In contrast, genes associated with dissimilatory sulfate reduction and oxidation, such as *Sat*, *AprA* and *DsrA*, increased in different LDPE treatments. By 60 d, the abundances of *CysCDHJN* and *Sir* increased in different LDPE treatments compared with CK, while *PAPSS* decreased in (Fig. S1D).

### Potential factors for LDPE-mediated impacts on CVW

**Fig. 6:**
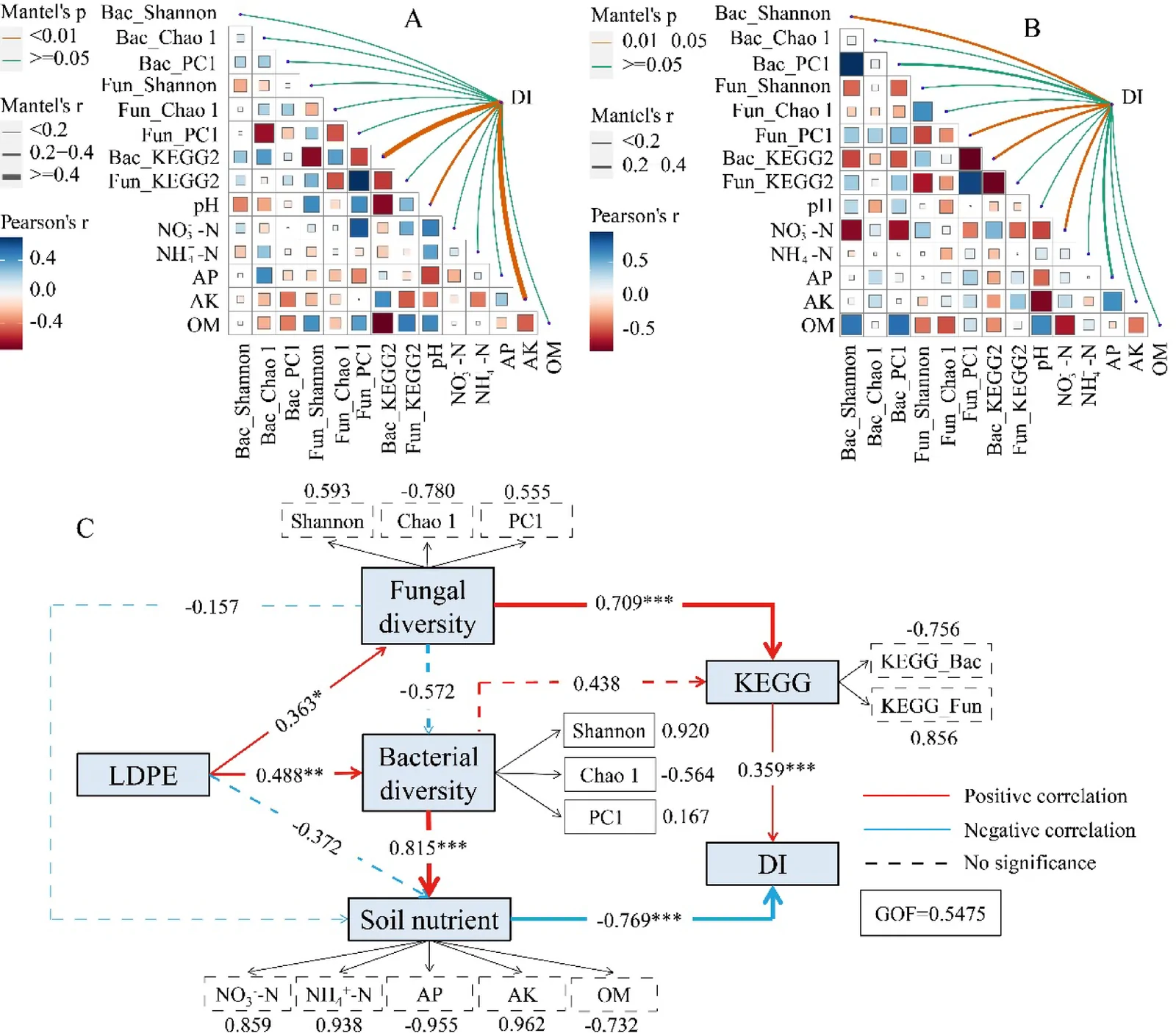
Mantel test of the correlation between environmental factors and the DI of verticillium wilt under different LDPE treatments at **A** 30 d and **B** 60d. **C** Partial least squares path modeling (PLS-PM) analysis for soil nutrients, microbial communities, and DI in different LDPE treatments. Red arrows indicate positive effects and blue arrows indicate negative effects. Wider arrows indicate greater effects. Dashed lines indicate no significant effect (*P* > 0.05). *Note*: GOF, goodness of fit

Mantel test was conducted to assess correlations among soil nutrients, α diversity and community structure (Bac_Shannon, Bac_Chao 1, Fun_Shannon, Fun_Chao 1, Bac_PC1 and Fun_PC1), β diversity of KEGG level 2 pathway (bacterial and fungal) and DI. At 30 d, DI showed significantly positive correlations with Bac_KEGG2 (r = 0.496, *P* = 0.002), pH (r = 0.269, *P* = 0.006), AK (r = 0.478, *P* = 0.001) (Fig. 6A). At 60 d, DI was significantly correlated with Bac_Shannon (r = 0.301, *P* = 0.013), Fun_PC1 (r = 0.282, *P* = 0.029), Bac_KEGG2 (r = 0.325, *P* = 0.011), NO_3_^−^-N (r = 0.396, *P* = 0.013) (Fig. 6B).

PLS-PM elucidates the interactions among LDPE treatments, soil nutrients (pH, NO_3_^−^-N, NH_4_^+^-N, AP, AK, OM), bacterial and fungal community diversities (Shannon, Chao 1 and PC1), KEGG pathways (bacterial and fungal), and DI (Fig. 6C). This model indicated that different LDPE treatments had significant direct positive effects on bacterial diversities (path coefficient = 0.363) and fungal diversities (path coefficient = 0.488). Bacterial and fungal diversities, in turn, showed significant positive effects on soil nutrients (path coefficient = 0.815) and KEGG (path coefficient = 0.709), respectively. Soil nutrients (path coefficient = − 0.769) and KEGG (path coefficient = 0.359) further exhibited significant effects on DI.

## Discussion

### Effect of microplastics treatments on plant health

Related studies have demonstrated that microplastics lead to significant changes in emergence rates, aboveground biomass, and root characteristics [25]. Bosker et al. [26] attributed the delay in seed germination to microplastic accumulation within testa pores, with physical obstruction proposed as the most plausible underlying mechanism. Wang et al. [27] reported that 1.0% PE and polybutylene succinate had a negative impact on soybean fresh weight. Yang et al. [9] observed that both biodegradable mulch film and PE significantly decreased rice height, dry weight of rice shoot and root after 120 d. Similarly, Li et al. [28] observed that polyethylene (PE) exerted an adverse effect on the plant height and culm diameter of soybean plants during the growth period. Furthermore, Wu et al. [29] documented that polystyrene microplastics caused a 10.62% reduction in the head rice yield of Y-liang-you 900 at harvest.

One possible explanation is that microplastic induce the formation of water-conducting channels, which greatly increases the rate of evaporation of soil moisture [30]. Previous research documented significant biomass reduction in cotton under plastic mulch, potentially attributed to microplastic-mediated inhibition of nitrogen alkaline hydrolysis [31]. This study also observed altered soil NO₃^−^-N availability under LDPE treatments, which may have contributed to the changes in plant growth performance.

Ren et al. [32] reported that the addition of 100 mg/kg PS combined with 1.0% degradable mulching film increased wheat plant height while simultaneously reducing total biomass. Our findings are consistent with this observation. The phenomenon may be attributed to compensatory mechanisms, where plants reallocate resources to mitigate the adverse effects of microplastic stress [33]. Similarly, although environmental stress limits the overall growth and biomass accumulation of *Leymus chinensis*, the plant shifts more energy toward stem growth-resulting in increased plant height to maintain essential morphological traits and potential reproductive output as a strategy for survival and propagation under adverse conditions [34]. We hypothesize that the observed reduction in biomass alongside increased plant height under 0.5–2.5% LDPE treatments may reflect a compensatory strategy, wherein plants enhance light capture through increased height to compensate for reduced biomass under stress.

At present, our knowledge regarding the effects of microplastics on soil-borne diseases is still largely incomplete. Cao et al. [35] reported that polystyrene nanoplastic and microplastic increased the incidence of bacterial wilt in tomato crops. Similarly, Zhang et al. [36] observed that high-density polyethylene (HDPE) increased the incidence of cotton *Fusarium* wilt by 33.3%, proposing that HDPE affects root-associated bacterial communities, interactions and functional metabolism in cotton. Oussama et al. [37] demonstrated that PE microplastics significantly enhance the virulence of *Fusarium solani*, resulting in a pronounced reduction in tomato root biomass and elongation. This effect was attributed to PE-induced sporulation as a fungal adaptive strategy to environmental stress, thereby intensifying pathogenic pressure on host plants [37]. Gkoutselis et al. [38] further revealed that microplastics enhance soil fungal pathogen fitness through nutrient provisioning and stress resistance augmentation, exacerbating plant disease risks. This study also found that microbial community structure and function, rather than the populations of *V. dahliae*, were key to the exacerbation of CVW (Fig. 8D). This is also supported by the observation that CK showed no disease at 30 d despite containing *V. dahliae*, whereas all LDPE treatments already displayed symptoms at the same time point, confirming that LDPE accelerated disease. This acceleration may be attributable to LDPE-induced disruption of the rhizosphere microbial balance, which is consistent with the view that soil-borne diseases arise from an imbalance between beneficial and pathogenic communities [39]. Recent studies have indicated that the relative abundances of beneficial bacteria, such as *Sphingomonas*, *Nocardioides*, *Gemmatirosa* and *Sphingobacterium*, were increased in soils with lower levels of CVW [22], which is similar conclusion to this study. Furthermore, plant growth and development are modulated by microbial nutrient provisioning through metabolic processes [40].

In the study, LDPE microplastics indirectly suppressed the function of soil bacteria in key metabolic processes, such as amino acids metabolic, carbohydrates metabolic, energy metabolic, signal transduction and membrane transport. Signal transduction and membrane transport processes are critical for cells to sense and respond to external environmental changes, thereby sustaining intracellular homeostasis.[41, 42]. Amino acid activity is positively correlated with pathogen resistance [43], while energy metabolism is closely linked to plant defensive responses [44], both processes serve as crucial regulators of plant pathogen defense mechanisms. Consistent with this findings, Zhao et al. [21] reported an inverse relationship between DI and functional gene abundance associated with energy metabolism and secondary metabolite biosynthesis pathways. The reduction in the abundance of these metabolic pathways may impair the ability of beneficial bacteria to supply adequate nutrients and energy for cotton growth and disease resistance.

In recent decades, the effects of soil nutrients availability on plant diseases have received increasing attention. Heck et al. [45] reported that soil phosphorus and potassium was negatively correlated with the severity of *Fusarium* wilt in banana. In this study, a significant reduction in AK content was observed in soils with aggravated disease severity at 30 d. In contrast, another study demonstrated that excessive phosphorus supply can increase plant susceptibility to pathogenic bacteria [46], a trend consistent with AP content observed at 60 d in this study. These variations may be attributed to differences in crops species and disease types. Although the correlation between soil nutrients and plant disease incidence is well-established, the dual effects of soil nutrients on disease development remain to be fully elucidated and thus warrant further investigation.

### Effect of microplastics treatments on soil nutrients

The NO_3_^−^-N content was influenced by exogenous addition of microplastics, which is consistent with the findings of previous reports. [47, 48]. Notably, the response of NO_3_^−^-N at 30 d was concentration-specific, with significant decreases under the 0.1% and 1.0% LDPE treatments but a significant increase under the 0.5% treatment, indicating that LDPE did not exert a simple dose-dependent effect on soil nitrogen cycling. A recent meta-analysis showed that the effects of microplastics on soil NO₃⁻-N are highly context-dependent, with both increases and decreases reported under different experimental conditions, depending on polymer type, exposure duration, soil properties and plant presence [49]. Compared with CK, the abundance of the key nitrification gene *amoA* showed a decreasing trend in the 0.1% and 1.0% LDPE treatments but an increasing trend in the 0.5% LDPE treatment at 30 d (Fig. S1A), which was consistent with the observed variation in soil NO_3_^−^-N. As ammonia oxidation is generally considered the rate-limiting step of nitrification [50], these results suggest that the differential responses of *amoA* across LDPE treatments may have contributed to the early changes in soil NO₃^−^-N. By 60 d, no significant differences in soil NO₃^−^-N were detected among treatments, suggesting that the early effects of LDPE on soil nitrate availability became less pronounced over time.

In addition, there was no significantly differences in NH_4_^+^-N content, which may be attributed to the balance between production and consumption of NH_4_^+^-N, the similar result was reported by Liu et al. [47]. Soil enzymes, derived from both microbial and plant sources, critically regulate biogeochemical cycling processes and energy transfer mechanisms in terrestrial ecosystems and are high sensitivity to environmental disturbances [51]. Moreover, MPs can modulate soil nitrogen dynamics by influencing the activities of key nitrogen-transforming enzymes, such as urease and β-N-acetylglucosaminidase [52].

Soil pH represents a major abiotic factors influencing soil chemical processes and microbial activity [53]. In this study, pH tended to be higher under LDPE treatments at 30 d, which is consistent with previous studies [54, 55]. The observed elevation in soil pH may be attributed to a reduction in soil buffering capacity, which arises from microplastic-induced alterations in soil composition and physicochemical properties [56]. Alternatively, microplastics-induced variation in soil pH could result from disturbance to soil microbial communities and their associated biogeochemical processes [57]. As an illustration, the elevation in soil pH induced by PE application has been attributed to improved soil aeration and altered functional activity of soil microbes, which promote NH_4_^+^-N production and H^+^ consumption [58]. Similarly, NH_4_^+^-N content tended to be higher under all LDPE treatments than CK at 30 d in this study. Furthermore, the incorporation of PE into soil may influence nitrogen cycling by upregulating key functional genes (*nifH*, *nirK*, *amoA*), potentially driving subsequent pH modifications through biogeochemical feedback mechanisms [59].

Regarding soil phosphorus (P) dynamics, available phosphorus (AP) content was significantly increased in 0.1%, 0.5%, and 1.0% LDPE treatments at 60 d, which may be attributed to the ability of microplastics to provide additional carbon sources and specific ecological niches for soil microbial communities, thereby stimulating phosphatase activity and enhancing phosphorus mobilization [60]. Consistently, Yi et al. [61] reported that both PE and PP increased phosphatase activity and elevated inorganic phosphorus levels. Furthermore, microplastics influence the activities of on urease, protease, and dehydrogenase, subsequently modifying phosphorus dynamics through perturbations in carbon and nitrogen cycling processes [62]. Doubtlessly, MPs could increase OM and total carbon content, given their inherent carbon-based polymeric composition [47]. Previous study has demonstrated that MPs contain substantial proportions of inert carbon compounds, accounting for over 90% of the composition, indicating their potential to function as direct carbon sources in soil environments [63]. It has been confirmed in this findings and is further supported by Shi et al. [64], who reported that 1.0% PE and PLA addition significantly increases dissolved organic carbon content. As reported by Yu et al. [61], application of 0.5% and 1.0% PE also led to a significant elevation in OM content, a response likely driven by the enhanced activities of peroxidase and fluorescein diacetate hydrolase under microplastic exposure, thereby facilitating the accumulation of dissolved organic compounds.

### Responses of microbial community composition under different LDPE treatments

Soil microbial communities play an essential role in sustaining the functional stability of soil ecosystems. Soil contamination, including microplastic pollution, can significantly alter the structure and composition of soil microbial communities [64]. It was found significant difference in bacterial and fungal community structure at 30 d and 60 d in this study. Compared with CK, the relative abundances of beneficial bacteria were significantly decreased, such as *Nitrospirae*, *Nocardioides*, *Devosia, Steroidobacter* and *Streptomyces*. *Nitrospirae* are nitrite-oxidizing bacteria that play a major role in nitrification in soils, and the lower its abundance, the lower NO_3_^−^-N content [65]. *Nocardioides* are capable of degrading organic pollutants, such as ibuprofen and atrazine [66, 67]. *Devosia* is rhizobacteria that can symbiotically fix nitrogen with legumes [68]. *Streptomyces* exert effective biocontrol effects against many fungal plant pathogens [69]. Xue et al. [70] reported that *Streptomyces* could efficiently inhibit the growth of *V. dahliae* and its conidia production.

In addition, compared with CK, there were more beneficial bacteria significantly reduced under LDPE treatments at 60d, such as *Bradyrhizobium*, *Sphingomonas*, *Microvirga*, *Mesorhizobium* and *Sphingosinicella.* Wu et al. [15] similarly observed a reduction in *Sphingomonas* abundance in soils amended with 2% PE microplastics after 60 d. Multiple *Bradyrhizobium* from host-specific symbiotic associations with legumes, facilitating atmospheric nitrogen fixation within root nodules [71]. Both *Microvirga* and *Bradyrhizobium* contribute to plants growth through nitrogen-fixation activities [72]. Certain *Sphingomonas* strains participate in nitrogen cycle processes, including nitrogen fixation and denitrification, and also aid in degrading soil contaminants [73]. Some beneficial microorganisms are more abundant in healthy soils, contributing to improved soil quality and inhibited soil pathogens. *Rhizophagus* which belongs to the mycorrhizal fungal and have beneficial effects on plant survival [74], was significantly decreased in our study (0.5%LDPE, 30 d). Furthermore, some *Aspergillus* and *Chaetomium*, reported as disease-preventing and growth-promoting microorganisms [75, 76], showed significantly decreased. Therefore, these results suggest that LDPE-induced disruption of beneficial bacterial communities in the cotton rhizosphere represents a key mechanism underlying the exacerbation of CVW.

### Relationships between soil nutrients and microbial communities

Soil physicochemical properties play a key role in shaping the microbial community [64]. Elucidating the relationships between soil factors and microbial diversity provides critical insights into microbe-plant-soil interactions. In this study, both bacterial and fungal community were significantly correlated with NO_3_^−^-N, NH_4_^+^-N, AP, AK and OM. Among these factors, NH_4_^+^-N was identified as the primary determinant influencing the microbiome in LDPE-amended soils. Yao et al. [77] reported that soil microbial community structure covered by plastic film was significantly correlated with AK, NH_4_^+^-N and pH. Similarly, Li et al. [78] demonstrated that microbial community abundance was closely linked to NO_3_^−^-N, NH_4_^+^-N and TN contents. Furthermore, some bacteria associated with the N cycle such as *Nitrospira*, *Sphingomonas*, *Microvirga* and *Devosia* may have an impact on soil N content [79, 80].

Soil physical and chemical properties can reshape microbial community, and thereby influence their functional potential [81]. Recent studies have reported genes of pathways such as microbial membrane transport and amino acid metabolism decreased in LDPE treatment [15]. Similarly, Fu et al. [82] also observed that PE significant suppressed several metabolic pathways in microorganisms, such as carbohydrate metabolism, amino acid metabolism and membrane transport. Since soil microorganisms often rely on metabolic activities to synthesize antibiotics and antibacterial proteins for disease suppression and plant growth promotion [83]. It was reported that decreased genes of KEGG pathway may contribute to inactive microbial activity. Therefore, it was inferred that limitations in the metabolic functions of beneficial microorganisms may contribute to the deterioration of soil health. Spearman correlations analysis revealed that predominant KEGG pathways (top 20) in bacterial community were correlated with NO_3_^−^-N, NH_4_^+^-N, AP, and AK contents (*P* < 0.05), while those in the fungal community were significantly associated with AK. The amino acid metabolism pathway is strongly linked to the degradation alanine, aspartate, and other carbohydrates, and the higher abundance of this functional content on MPs may potentially affect the carbon and nitrogen content in the soil [84]. The addition of microplastics reduced amino acid, carbohydrate, and energy metabolism, likely due to microplastic-induced alterations in soil nutrients and moisture (e.g., nitrogen fixation) [85]. In support of this, Lu et al. [86] observed a positive correlation between soil inorganic nitrogen content and both carbon and lipid metabolism, consistent with the this findings. Furthermore, alterations in soil microbial KEGG pathway often correspond to changes in enzyme activity [87]. Soil enzyme activity serves as a direct indicator of microbial metabolic demands and nutrient availability, where downregulation of amino acid, carbohydrate, and lipid metabolism may lead to reduced activity of enzymes involved in carbon, nitrogen, and phosphorus cycling [88].

## Conclusions

LDPE microplastic inhibits cotton growth, reduces plant fresh biomass, and significantly exacerbates the occurrence of CVW, with no significant increase in the abundance of *V. dahliae*. Metagenomics analysis revealed that LDPE caused significant changes in soil microbial community structure and function at different periods, with bacterial KEGG pathways significantly correlated with NO_3_^−^-N, NH_4_^+^-N, AP, AK and OM. Further analysis revealed that the abundances of key genes associated with nitrogen cycling (*NapB* and *NrfAH*) and sulfur cycling (*CysCDH* and *Sir*) decreased in 1.0% and 2.5% LDPE treatments at 30 d, while most genes (*NirBD*, *NrfAH*, *CysCDIJN* and *Sir*) increased in abundance by 60 d. Meanwhile, the PLS-PM results demonstrated that LDPE-induced changes in the structure and function of fungal community were significantly positively correlated with DI, while such changes in bacterial community were significantly positively correlated with soil nutrients. These findings collectively indicate that LDPE directly influences the structure and function of cotton rhizosphere microbiota, ultimately impairing plant health.

## Supplementary Information

Below is the link to the electronic supplementary material.

Supplementary Material 1

## Data Availability

The raw sequence data generated in this study have been deposited in the NCBI Sequence Read Archive (SRA) under the BioProject accession number PRJNA1417499.
