# Supplementary material for: Effect of low-density polyethylene microplastics on incidence of cotton Verticillium wilt and the structure and function of rhizosphere microbial community
Source: Environ Microbiome. 2026 Aug 1;21:128. doi: 10.1186/s40793-026-00943-2 (PMC13718038; doi:10.1186/s40793-026-00943-2)
Supplement: Supplementary file 1 [file 40793_2026_943_MOESM1_ESM.docx]

**Supplementary information: Fig. S1** Diagrams of nitrogen and sulfur pathways and the difference in abundance of genes under different LDPE treatments. Nitrogen pathways at (A) 30 d and (B) 60 d, sulfur pathways at (C) 30 d and (D) 60 d. The order of percentages in brackets is as follows: control (CK) and 0.1% LDPE, CK and 0.5% LDPE, CK and 1.0% LDPE, CK and 2.5% LDPE. The percentage expressed in red or green represents the increase or decrease of the corresponding gene abundance than CK, respectively. Different color blocks represent different paths.

**Supplementary information: Table S1** Overview of the assembled in soil microorganism metagenome under different LDPE treatment.

| samples | raw reads | raw base (bp) | clean reads | clean base (bp) | percent in raw reads (%) | percent in raw bases (%) | contigs | contigs bases (bp) | n50 (bp) | Max (bp) |
| --- | --- | --- | --- | --- | --- | --- | --- | --- | --- | --- |
| CK1_30d | 66392364 | 10025246964 | 64801648 | 9.77e+09 | 97.60407 | 97.46491 | 432631 | 229797884 | 532 | 20643 |
| CK2_30d | 56418994 | 8519268094 | 55018552 | 8.29e+09 | 97.51778 | 97.34262 | 367990 | 190051821 | 513 | 28571 |
| CK3_30d | 51875370 | 7833180870 | 51000952 | 7.69e+09 | 98.31439 | 98.16631 | 319801 | 160759698 | 500 | 7067 |
| 0.1%1_30d | 71073736 | 10732134136 | 69700752 | 1.05e+10 | 98.06823 | 97.90813 | 544290 | 276904932 | 504 | 22241 |
| 0.1%2_30d | 54918294 | 8292662394 | 53576080 | 8.07e+09 | 97.55598 | 97.34972 | 382635 | 184550797 | 477 | 10678 |
| 0.1%3_30d | 57009126 | 8608378026 | 55864762 | 8.42e+09 | 97.99267 | 97.84485 | 394960 | 197263182 | 496 | 27132 |
| 0.5%1_30d | 61537452 | 9292155252 | 60299318 | 9.09e+09 | 97.988 | 97.82965 | 461493 | 232059964 | 500 | 9892 |
| 0.5%2_30d | 66602684 | 10057005284 | 65339622 | 9.85e+09 | 98.10359 | 97.95149 | 483549 | 246908100 | 508 | 9057 |
| 0.5%3_30d | 58472358 | 8829326058 | 57315248 | 8.64e+09 | 98.0211 | 97.86571 | 419688 | 212505949 | 504 | 9180 |
| 1.0%1_30d | 60410066 | 9121919966 | 59183256 | 8.92e+09 | 97.9692 | 97.81152 | 447020 | 223323030 | 495 | 9305 |
| 1.0%2_30d | 54808704 | 8276114304 | 53821908 | 8.11e+09 | 98.19956 | 98.04739 | 375325 | 185608589 | 491 | 7146 |
| 1.0%3_30d | 49977358 | 7546581058 | 48992684 | 7.39e+09 | 98.02976 | 97.87441 | 309615 | 154410874 | 500 | 7503 |
| 2.5%1_30d | 48696998 | 7353246698 | 47701884 | 7.19e+09 | 97.95652 | 97.80319 | 292158 | 144712966 | 495 | 7179 |
| 2.5%2_30d | 59071702 | 8919827002 | 57914968 | 8.73e+09 | 98.04181 | 97.82884 | 406815 | 202496978 | 493 | 8308 |
| 2.5%3_30d | 54069414 | 8164481514 | 52966032 | 7.98e+09 | 97.95932 | 97.76872 | 344899 | 174456393 | 506 | 18966 |
| CK_1_60d | 54979628 | 8301923828 | 54011824 | 8.14e+09 | 98.2397 | 98.10434 | 347979 | 171828128 | 492 | 10555 |
| CK_2_60d | 56539780 | 8537506780 | 55667762 | 8.39e+09 | 98.45769 | 98.3111 | 403507 | 197374478 | 487 | 7765 |
| CK_3_60d | 59607864 | 9000787464 | 58785138 | 8.86e+09 | 98.61977 | 98.47594 | 434138 | 211781654 | 483 | 6466 |
| 0.1%1_60d | 60122778 | 9078539478 | 59128484 | 8.92e+09 | 98.34623 | 98.20839 | 422705 | 208454543 | 491 | 7465 |
| 0.1%2_60d | 58619116 | 8851486516 | 57773546 | 8.71e+09 | 98.55752 | 98.42327 | 400659 | 199935238 | 498 | 7100 |
| 0.1%3_60d | 57531042 | 8687187342 | 56525492 | 8.52e+09 | 98.25216 | 98.12139 | 383126 | 189579004 | 495 | 5770 |
| 0.5%1_60d | 53844044 | 8130450644 | 52979032 | 7.99e+09 | 98.39349 | 98.25542 | 337239 | 166084965 | 492 | 8141 |
| 0.5%2_60d | 61019084 | 9213881684 | 60015062 | 9.05e+09 | 98.35458 | 98.21138 | 408080 | 202663793 | 495 | 11081 |
| 0.5%3_60d | 60511174 | 9137187274 | 59633522 | 8.99e+09 | 98.5496 | 98.41186 | 396915 | 193237912 | 484 | 9390 |
| 1.0%1_60d | 56737434 | 8567352534 | 55710032 | 8.4e+09 | 98.1892 | 98.04067 | 344669 | 167173529 | 485 | 8107 |
| 1.0%2_60d | 61922540 | 9350303540 | 60984936 | 9.19e+09 | 98.48584 | 98.33351 | 448440 | 218126161 | 482 | 10944 |
| 1.0%3_60d | 60901234 | 9196086334 | 59623564 | 8.99e+09 | 97.90206 | 97.75564 | 383424 | 189099217 | 493 | 5730 |
| 2.5%1_60d | 59632734 | 9004542834 | 58718010 | 8.85e+09 | 98.46607 | 98.3271 | 385486 | 188588782 | 489 | 8880 |
| 2.5%2_60d | 53650522 | 8101228822 | 52787240 | 7.96e+09 | 98.39092 | 98.23961 | 328024 | 158608636 | 483 | 16870 |
| 2.5%3_60d | 58170646 | 8783767546 | 57087022 | 8.61e+09 | 98.13716 | 97.99523 | 369980 | 183134670 | 494 | 11416 |
| sum/average | 1745124240 | 2.63514E+11 | 1712928332 | 2.58e+11 | 98.15547 | 98.00241 | 11777240 | 5861481867 | 495.2333 | 338548 |

Note: bp, base pairs.

**Supplementary information: Table S2** Difference of bacterial community in different LDPE treatments at 30 d (Phylum level).

| name | CK-mean (%) | CK-SD (%) | 0.1%-mean (%) | 0.1%-SD (%) | 0.5%-mean (%) | 0.5%-SD (%) | 1.0%-mean (%) | 1.0%-SD (%) | 2.5%-mean (%) | 2.5%-SD (%) |
| --- | --- | --- | --- | --- | --- | --- | --- | --- | --- | --- |
| p__Candidatus_Eremiobacteraeota | 0.3791 | 0.004534 | 0.3031 | 0.005188 | 0.3677 | 0.009449 | 0.3511 | 0.004605 | 0.3943 | 0.002003 |
| p__Candidatus_Saccharibacteria | 0.04367 | 0.003394 | 0.04532 | 0.003403 | 0.05593 | 0.007983 | 0.08009 | 0.006174 | 0.1182 | 0.008033 |
| p__Candidatus_Raymondbacteria | 0.01673 | 0.00043 | 0.01089 | 0.0007552 | 0.009668 | 0.00079 | 0.008984 | 0.000961 | 0.008783 | 0.000409 |
| p__Bacteroidetes | 1.244 | 0.005983 | 1.199 | 0.02793 | 1.49 | 0.1676 | 1.448 | 0.0521 | 1.341 | 0.1026 |
| p__Calditrichaeota | 0.0472 | 0.000802 | 0.0419 | 0.001119 | 0.03799 | 0.002367 | 0.03741 | 0.000496 | 0.0389 | 0.001411 |
| p__Balneolaeota | 0.01442 | 0.000333 | 0.01394 | 0.000725 | 0.01296 | 0.000614 | 0.01314 | 0.000577 | 0.01158 | 0.000208 |
| p__Candidatus_Nomurabacteria | 0.00156 | 2.54e-05 | 0.002083 | 0.0002257 | 0.002038 | 0.000249 | 0.002349 | 0.000268 | 0.003122 | 0.000228 |
| p__Verrucomicrobia | 1.104 | 0.02652 | 1.034 | 0.04721 | 0.9682 | 0.0626 | 0.9627 | 0.03018 | 0.8532 | 0.01481 |
| p__Spirochaetes | 0.04782 | 0.001143 | 0.03928 | 0.001814 | 0.03465 | 0.003399 | 0.0339 | 0.000463 | 0.03495 | 0.000109 |
| p__Lentisphaerae | 0.03175 | 0.002084 | 0.0293 | 0.0005568 | 0.02403 | 0.001457 | 0.02497 | 0.001157 | 0.02565 | 0.001323 |
| p__Fibrobacteres | 0.4969 | 0.00743 | 0.3279 | 0.02411 | 0.2738 | 0.01402 | 0.2731 | 0.01051 | 0.2898 | 0.01916 |
| p__Ignavibacteriae | 0.03829 | 0.000804 | 0.03413 | 0.00077 | 0.03322 | 0.000528 | 0.03266 | 0.001159 | 0.03198 | 0.000758 |
| p__Acidobacteria | 16.79 | 0.04607 | 16.15 | 0.3172 | 16.88 | 0.5194 | 16.41 | 0.2653 | 15.39 | 0.2568 |
| p__Elusimicrobia | 0.04357 | 0.000914 | 0.04676 | 0.003729 | 0.04138 | 0.00138 | 0.04151 | 0.001847 | 0.04063 | 0.001221 |
| p__Candidatus_Rokubacteria | 1.487 | 0.02546 | 1.591 | 0.01892 | 1.438 | 0.0592 | 1.469 | 0.06029 | 1.572 | 0.03217 |
| p__Candidatus_Dadabacteria | 0.0293 | 0.002026 | 0.02661 | 0.001519 | 0.02604 | 0.000295 | 0.02572 | 0.000811 | 0.02367 | 0.001158 |
| p__candidate_division_CPR2 | 7.17e-05 | 2.86e-05 | 4.23e-05 | 7.41e-06 | 0.000121 | 1.40e-05 | 0.000161 | 0.000133 | 0.00026 | 7.75e-05 |
| p__Candidatus_Woykebacteria | 0.00077 | 0.000132 | 0.0009379 | 0.0001374 | 0.001162 | 0.000121 | 0.00082 | 0.000129 | 0.001085 | 7.42e-05 |
| p__Candidatus_Parcubacteria | 0.004084 | 0.000772 | 0.006421 | 0.000857 | 0.005593 | 0.000484 | 0.005144 | 0.000501 | 0.006486 | 0.00085 |
| p__Candidatus_Cloacimonetes | 0.04398 | 0.001461 | 0.0469 | 0.0007084 | 0.05027 | 0.001188 | 0.05048 | 0.000986 | 0.04969 | 0.003022 |
| p__Candidatus_Latescibacteria | 0.03493 | 0.001749 | 0.03088 | 0.001567 | 0.02851 | 0.000714 | 0.03031 | 0.00204 | 0.02973 | 0.001236 |
| p__Fusobacteria | 0.002664 | 3.87e-05 | 0.001658 | 0.0001341 | 0.001795 | 0.000315 | 0.002464 | 0.000394 | 0.001773 | 0.000172 |
| p__Chloroflexi | 5.264 | 0.03083 | 5.312 | 0.09657 | 5.51 | 0.05123 | 5.419 | 0.09408 | 5.481 | 0.05619 |
| p__Proteobacteria | 41.71 | 0.331 | 41.83 | 0.6004 | 41.63 | 0.3197 | 43.37 | 0.04294 | 42.08 | 0.09046 |
| p__Synergistetes | 0.00432 | 0.000192 | 0.003896 | 0.000147 | 0.003348 | 0.000435 | 0.003753 | 0.000275 | 0.003594 | 0.000505 |

Note: SD, standard deviation.

**Supplementary information: Table S3** Difference of bacterial community in different LDPE treatments at 60 d (Phylum level).

| name | CK-mean (%) | CK-SD (%) | 0.1%-mean (%) | 0.1%-SD (%) | 0.5%-mean (%) | 0.5%-SD (%) | 1.0%-mean (%) | 1.0%-SD (%) | 2.5%-mean (%) | 2.5%-SD (%) |
| --- | --- | --- | --- | --- | --- | --- | --- | --- | --- | --- |
| p__Candidatus_Gottesmanbacteria | 0.007328 | 0.000471 | 0.008651 | 0.001103 | 0.01206 | 0.000237 | 0.01453 | 0.00042 | 0.01585 | 0.000174 |
| p__Candidatus_Collierbacteria | 0.002988 | 0.000127 | 0.006232 | 0.001026 | 0.008797 | 0.00152 | 0.01337 | 0.001913 | 0.005477 | 0.00076 |
| p__Acidobacteria | 20.72 | 0.4112 | 19.96 | 0.2824 | 19.15 | 0.1574 | 18.83 | 0.1532 | 19.57 | 0.1118 |
| p__Cyanobacteria | 0.5005 | 0.004789 | 0.4911 | 0.004042 | 0.4822 | 0.01024 | 0.4735 | 0.00277 | 0.5062 | 0.009469 |
| p__Candidatus_Rokubacteria | 1.226 | 0.01678 | 1.211 | 0.0454 | 1.326 | 0.01253 | 1.385 | 0.03108 | 1.324 | 0.05673 |
| p__Fibrobacteres | 0.243 | 0.002121 | 0.1952 | 0.01119 | 0.2272 | 0.0221 | 0.1894 | 0.01422 | 0.1729 | 0.01669 |
| p__Proteobacteria | 41.21 | 0.212 | 42.29 | 0.3515 | 43.02 | 0.7381 | 42.98 | 0.3235 | 43.96 | 0.8124 |
| p__Candidatus_Shapirobacteria | 0.000231 | 3.31e-05 | 0.000314 | 3.26e-05 | 0.000624 | 0.000228 | 0.000437 | 8.66e-05 | 0.00049 | 0.000152 |
| p__Candidatus_Latescibacteria | 0.0361 | 0.000631 | 0.03298 | 0.0012 | 0.03318 | 0.000718 | 0.0319 | 0.000745 | 0.03422 | 0.00102 |
| p__Nitrospirae | 0.9402 | 0.04084 | 0.8075 | 0.04954 | 0.8009 | 0.004561 | 0.7618 | 0.005289 | 0.8972 | 0.03051 |
| p__Candidatus_Daviesbacteria | 0.001931 | 1.27e-05 | 0.002248 | 0.000192 | 0.001716 | 4.21e-05 | 0.001509 | 0.000368 | 0.002172 | 0.000311 |
| p__Candidatus_Woesebacteria | 0.002895 | 0.000191 | 0.00356 | 0.000206 | 0.003128 | 6.68e-05 | 0.003362 | 0.000499 | 0.004528 | 0.000303 |
| p__Planctomycetes | 1.693 | 0.0432 | 1.754 | 0.05283 | 1.68 | 0.01239 | 1.634 | 0.01339 | 1.739 | 0.04363 |
| p__Armatimonadetes | 0.1692 | 0.002451 | 0.1657 | 0.007825 | 0.1505 | 0.003704 | 0.145 | 0.005489 | 0.1615 | 0.008119 |
| p__Candidatus_Eremiobacteraeota | 0.1938 | 0.005003 | 0.1761 | 0.006181 | 0.1853 | 0.008404 | 0.1659 | 0.004539 | 0.1961 | 0.01331 |
| p__Chloroflexi | 6.697 | 0.04817 | 6.738 | 0.08799 | 6.391 | 0.1762 | 6.73 | 0.04399 | 6.527 | 0.02989 |
| p__Candidatus_Levybacteria | 0.002412 | 0.00016 | 0.003195 | 0.000142 | 0.002848 | 0.000226 | 0.003442 | 0.000331 | 0.003355 | 0.000368 |
| p__Candidatus_Eisenbacteria | 0.09987 | 0.001822 | 0.1009 | 0.000947 | 0.09813 | 0.001793 | 0.1051 | 0.003123 | 0.1045 | 0.001283 |
| p__Candidatus_Cloacimonetes | 0.04186 | 0.001555 | 0.04289 | 0.001493 | 0.03903 | 0.000724 | 0.04033 | 0.000991 | 0.04311 | 0.000789 |
| p__Candidatus_Tectomicrobia | 0.08609 | 0.006926 | 0.08231 | 0.004101 | 0.0795 | 0.002699 | 0.07741 | 0.003101 | 0.08904 | 0.002609 |
| p__Verrucomicrobia | 1.154 | 0.02139 | 1.099 | 0.04575 | 1.088 | 0.03334 | 1.03 | 0.02769 | 1.103 | 0.03812 |

Note: SD, standard deviation.

**Supplementary information: Table S4** Difference of fungal community in different LDPE treatments at 30 d (Phylum level)**.**

| name | CK-mean (%) | CK-SD (%) | 0.1%-mean (%) | 0.1%-SD (%) | 0.5%-mean (%) | 0.5%-SD (%) | 1.0%-mean (%) | 1.0%-SD (%) | 2.5%-mean (%) | 2.5%-SD (%) |
| --- | --- | --- | --- | --- | --- | --- | --- | --- | --- | --- |
| p__Ascomycota | 50.68 | 0.9049 | 49.23 | 2.592 | 53.7 | 0.771 | 51.82 | 3.507 | 56.93 | 3.864 |
| p__Mucoromycota | 34.7 | 1.899 | 38.37 | 2.38 | 30.34 | 1.761 | 33.46 | 3.469 | 29.79 | 3.309 |
| p__Basidiomycota | 9.383 | 0.7964 | 8.001 | 0.3379 | 9.734 | 1.207 | 8.845 | 0.37 | 8.813 | 0.8012 |
| p__Zoopagomycota | 2.001 | 0.609 | 1.887 | 0.6844 | 3.025 | 0.7434 | 2.871 | 0.6668 | 2.598 | 0.9955 |
| p__Chytridiomycota | 2.801 | 0.2605 | 2.488 | 0.6911 | 2.27 | 0.2155 | 2.634 | 0.7355 | 1.6 | 0.5676 |
| p__unclassified_k__Fungi | 0.08463 | 0.07564 | 0 | 0 | 0.5699 | 0.2622 | 0.2923 | 0.2613 | 0.209 | 0.2217 |
| p__Blastocladiomycota | 0.3487 | 0.3071 | 0.02967 | 0.05139 | 0.2212 | 0.2201 | 0 | 0 | 0.06613 | 0.1145 |
| p__Microsporidia | 0 | 0 | 0 | 0 | 0.1314 | 0.2275 | 0.08274 | 0.1433 | 0 | 0 |

Note: SD, standard deviation.

**Supplementary information: Table S5** Difference of fungal community in different LDPE treatments at 60 d (Phylum level)**.**

| name | CK-mean (%) | CK-SD (%) | 0.1%-mean (%) | 0.1%-SD (%) | 0.5%-mean (%) | 0.5%-SD (%) | 1.0%-mean (%) | 1.0%-SD (%) | 2.5%-mean (%) | 2.5%-SD (%) |
| --- | --- | --- | --- | --- | --- | --- | --- | --- | --- | --- |
| p__Mucoromycota | 49.73 | 2.687 | 48.24 | 3.487 | 43.68 | 4.255 | 47.3 | 5.127 | 50.15 | 4.917 |
| p__Ascomycota | 40.52 | 2.903 | 40.79 | 2.868 | 43.33 | 4.309 | 40.05 | 3.03 | 39.11 | 3.881 |
| p__Basidiomycota | 6.555 | 0.4475 | 7.748 | 0.5267 | 8.737 | 0.8727 | 9.46 | 1.567 | 7.69 | 0.9116 |
| p__Zoopagomycota | 1.508 | 0.1014 | 1.86 | 0.3404 | 2.002 | 0.581 | 1.421 | 0.2062 | 1.487 | 0.1717 |
| p__Chytridiomycota | 1.509 | 0.05891 | 1.159 | 0.3094 | 2.254 | 0.1588 | 1.615 | 0.5792 | 1.192 | 0.2814 |
| p__unclassified_k__Fungi | 0.1715 | 0.2237 | 0.05769 | 0.05104 | 0 | 0 | 0.1014 | 0.1022 | 0.2159 | 0.1255 |
| p__Blastocladiomycota | 0 | 0 | 0.06912 | 0.1197 | 0 | 0 | 0 | 0 | 0.1557 | 0.2697 |
| p__Microsporidia | 0 | 0 | 0.0801 | 0.0695 | 0 | 0 | 0.05573 | 0.09652 | 0 | 0 |

Note: SD, standard deviation.

**Supplementary information: Table S6** LDPE effects on the relative abundances of KEGG level 2 pathways in bacterial and fungal communities.

| time | microbial community | pathway | treatments | | | | |
| --- | --- | --- | --- | --- | --- | --- | --- |
|  |  |  | CK | 0.1% | 0.5% | 1.0% | 2.5% |
| 30 d | bacteria | global and overview maps | 5.421846 | 5.687569 | 5.96286 | 5.188247 | 5.069966 |
|  |  | carbohydrate metabolism | 1.870327 | 1.963258 | 2.078343 | 1.800561 | 1.737339 |
|  |  | amino acid metabolism | 1.736283 | 1.8286 | 1.91067 | 1.665885 | 1.633292 |
|  |  | energy metabolism | 1.264329 | 1.334822 | 1.4398 | 1.246873 | 1.195674 |
|  |  | metabolism of cofactors and vitamins | 0.905138 | 0.948177 | 0.975797 | 0.847414 | 0.844637 |
|  |  | membrane transport | 0.872081 | 0.929991 | 0.994553 | 0.86074 | 0.818972 |
|  |  | cellular community - prokaryotes | 0.741141 | 0.791994 | 0.838131 | 0.72691 | 0.692786 |
|  |  | signal transduction | 0.689794 | 0.710711 | 0.742686 | 0.651132 | 0.627392 |
|  | fungi | global and overview maps | 2.865119 | 2.431678 | 1.76315 | 1.741111 | 2.865119 |
|  |  | energy metabolism | 1.43256 | 0.83015 | 0.45548 | 0.506906 | 1.43256 |
|  |  | carbohydrate metabolism | 0.675874 | 0.866882 | 0.514252 | 0.609756 | 0.675874 |
|  |  | nervous system | 1.153394 | 0.492213 | 0.47752 | 0.345284 | 1.153394 |
|  |  | neurodegenerative disease | 1.168087 | 0.499559 | 0.330591 | 0.279165 | 1.168087 |
|  |  | environmental adaptation | 1.168087 | 0.506906 | 0.301205 | 0.279165 | 1.168087 |
|  |  | amino acid metabolism | 0.558331 | 0.47752 | 0.426095 | 0.337937 | 0.558331 |
|  |  | cancer: overview | 0.359976 | 0.426095 | 0.308551 | 0.367323 | 0.359976 |
| 60d | bacteria | global and overview maps | 5.343659 | 5.594167 | 5.476493 | 5.556611 | 5.219796 |
|  |  | carbohydrate metabolism | 1.886486 | 1.967126 | 1.898987 | 1.920781 | 1.823889 |
|  |  | amino acid metabolism | 1.705895 | 1.792448 | 1.75552 | 1.78653 | 1.67051 |
|  |  | energy metabolism | 1.289969 | 1.34392 | 1.281811 | 1.298272 | 1.243286 |
|  |  | membrane transport | 0.888545 | 0.926287 | 0.888849 | 0.902187 | 0.864608 |
|  |  | metabolism of cofactors and vitamins | 0.864353 | 0.904775 | 0.899742 | 0.92227 | 0.843933 |
|  |  | cellular community - prokaryotes | 0.755105 | 0.788536 | 0.756629 | 0.768685 | 0.743214 |
|  |  | signal transduction | 0.684569 | 0.722729 | 0.70816 | 0.71373 | 0.679326 |
|  | fungi | global and overview maps | 4.54501 | 4.73072 | 3.469847 | 3.098426 | 4.271332 |
|  |  | energy metabolism | 2.24807 | 2.443554 | 1.329293 | 1.153357 | 2.355586 |
|  |  | environmental adaptation | 1.690939 | 1.99394 | 0.889454 | 0.703744 | 2.228521 |
|  |  | neurodegenerative disease | 1.632294 | 1.964617 | 0.821034 | 0.723292 | 2.101456 |
|  |  | nervous system | 1.485681 | 1.642068 | 0.840583 | 0.576679 | 1.964617 |
|  |  | carbohydrate metabolism | 1.534552 | 1.143583 | 1.104486 | 1.270648 | 0.781937 |
|  |  | signal transduction | 0.918776 | 0.879679 | 0.987196 | 0.518033 | 0.781937 |
|  |  | transport and catabolism | 0.576679 | 0.596227 | 0.576679 | 0.410517 | 0.654872 |

**Supplementary information: Table S7** Monte Carlo permutation test of soil nutrients on microbial community composition.

| microbial communities | soil nutrient | rda1 | rda2 | r2 | p-values |
| --- | --- | --- | --- | --- | --- |
| bacteria | pH | 0.35433 | 0.93512 | 0.2835 | 0.018 |
|  | NO_3_^-^-N | 0.09786 | 0.99520 | 0.5763 | 0.001 |
|  | NH_4_^+^-N | 0.99176 | 0.12814 | 0.8711 | 0.001 |
|  | AP | -0.57150 | -0.82060 | 0.7763 | 0.001 |
|  | AK | 0.47066 | -0.88231 | 0.7306 | 0.001 |
|  | OM | -0.04569 | 0.99896 | 0.4831 | 0.001 |
| fungi | pH | 0.76913 | 0.63909 | 0.0204 | 0.743 |
|  | NO_3_^-^-N | 0.93919 | 0.34339 | 0.5225 | 0.001 |
|  | NH_4_^+^-N | -0.51820 | 0.85526 | 0.7861 | 0.001 |
|  | AP | -0.04370 | -0.99904 | 0.5904 | 0.001 |
|  | AK | -0.98978 | 0.14260 | 0.6456 | 0.001 |
|  | OM | 0.99998 | -0.00681 | 0.2184 | 0.001 |

Note: NO₃⁻-N, nitrate nitrogen; NH₄⁺-N, ammonium nitrogen; AP, available phosphorus; AK, available potassium; OM, organic matter.
